# A Protocol for the Development and Validation of the Postpartum Specific Anxiety Scale—Preterm Birth [PSAS‐PTB] and the Postpartum Specific Anxiety Scale—Neonatal Intensive Care Unit [PSAS‐NICU]

**DOI:** 10.1002/mpr.70032

**Published:** 2025-08-09

**Authors:** Semra Worrall, Paul Christiansen, Asma Khalil, Victoria Fallon, Sergio A. Silverio

**Affiliations:** ^1^ Department of Psychology Institute of Population Health University of Liverpool Liverpool UK; ^2^ Fetal Medicine Unit Liverpool Women's NHS Foundation Trust Liverpool UK; ^3^ Fetal Medicine Unit St. George's University Hospitals NHS Foundation Trust University of London London UK; ^4^ Department of Women & Children's Health School of Life Course & Population Sciences King's College London London UK

**Keywords:** cognitive interviews, NICU, postpartum anxiety, preterm birth, psychometrics, stakeholder engagement

## Abstract

**Objectives:**

To describe the development and the methodology for validation of a new scale for postpartum anxiety for mothers of preterm infants, and a ‘Velcro’ sub‐scale of the Postpartum Specific Anxiety Scale for use with mothers who have had infants admitted to the Neonatal Intensive Care Unit.

**Methods:**

We undertook three forms of iterative psychometric development: (1) Patient and public involvement and engagement discussions with key clinical, academic, and lay stakeholders to understand the needs for modifying the Postpartum Specific Anxiety Scale—Research Short Form for use in this population; (2) Expert panel ratings with clinical and academic stakeholders; and (3) Cognitive interviews with mothers to ensure items were relevant, comprehensive, and understandable. Planned studies must ensure the psychometric properties of these two new scales.

**Results:**

Patient and Public Involvement and Engagement discussions identified clear avenues for modification of the PSAS‐RSF, but the need for an additional NICU‐specific scale was clear. Experts rated the new items highly on their relevance. Cognitive interviews further ensured that items were well understood and that meaning was being interpreted in the intended manner. Only minor changes to the scales were implemented after each change.

**Conclusions:**

This is the first study to describe the process of developing and the subsequent proposed validation of postpartum‐specific tools for use with mothers of preterm infants and those with infants in the Neonatal Intensive Care Unit. Clear avenues have been identified for the validation and implementation of both measures.

## 1 Introduction

### 1.1 Postpartum Anxiety

Effective and accurate measurement of perinatal mental health conditions are vital to enable appropriate intervention. Estimates suggest up to 40% of women go on to develop postpartum anxiety (Field 2017), with lifetime costs reaching over £30,000 per woman, and the majority of this impact is on children (Bauer et al. 2016). Much of the research exploring anxiety in the period after birth has been limited by the use of generalised measures originally validated for use in general adult populations, such as the State Trait Anxiety Inventory (STAI; Spielberger 1983) and GAD‐7 (Spitzer et al. 2006), which have been extrapolated for use in a perinatal population. This is problematic for several reasons. Firstly, they were not validated for use during the postpartum period which means scores may be inaccurate. For example, the STAI features items such as *‘I feel rested’,* but sleeping difficulties are common in the context of new parenthood (Baglioni et al. 2022), irrespective of anxiety. Additionally, they do not address domains specific to a childbearing context, such as concerns over infant care, feeding, and the adjustment to motherhood, which are known to be significant sources of anxiety (Christl et al. 2013; Fallon et al. 2016; Hoff et al. 2019). This may lead to an underestimation of anxiety diagnoses in this population.

In response to the lack of appropriate measurement tools, the Postpartum Specific Anxiety Scale (PSAS; Fallon et al. 2016) was created. The 51‐item tool measures concern about attachment to the infant, routine care, health and accidental harm to the infant, and changes to relationships and finances after the baby's birth. Since then, a 12‐item Research Short Form for use during crises (PSAS‐RSF‐C; Silverio et al. 2021), and a 16‐item Research Short Form (PSAS‐RSF; Davies et al. 2021) have been developed and validated. The PSAS and its derivatives have well‐evidenced validity and reliability, and have shown to be better predictors of maternal and infant outcomes (Fallon et al. 2018) compared to generalised measures.

Further global work has been conducted to produce validated translations of the PSAS and its derivatives in French (PSAS‐FR; Infante‐Gil et al. 2022), Italian (PSAS‐IT; Ionio et al. 2023), Spanish (PSAS‐ES; Costas‐Ramón et al. 2023), Brazilian Portuguese (PSAS‐BR; Souza Torres de Araújo et al. 2024), Persian (PSAS‐IR; Hasanzadeh et al. 2021; PSAS‐IR‐RSF; Mashayekh‐Amiri, Jafarabadi, Davies, et al. 2023; PSAS‐IR‐RSF‐C, Mashayekh‐Amiri, Jafarabadi, Montazeri, et al. 2023), Chinese (PSAS‐CN; Xu et al. 2021), and Jordanian Arabic (PSAS‐JO‐RSF; Hijazi et al. 2024). Ongoing translations are currently being undertaken in Germany, The Netherlands, Portugal, Greece, Slovakia, Croatia, Sweden, Myanmar, India, Egypt, Czechia, and Türkiye, as well as being used in approximately 40 countries as part of research and clinical practice.

### 1.2 The Need to Investigate Anxiety in High‐Risk Populations

Preterm birth and the experiences of mothers with babies in the Neonatal Intensive Care Unit [NICU] remains under‐researched. Estimates suggest approximately 8% of infants in the UK are born prematurely (< 37 weeks' gestation; ONS 2024). Whilst in some cases the birth is medically indicated because it is either safer for the baby or the mother, in most cases preterm birth is spontaneous in women with no known risk factors (Vogel et al. 2018). This creates a problem for clinicians because despite increasing research into the correlates and predictors of preterm birth (Carter et al. 2020; Reicher et al. 2021), the causes are relatively unknown (Goldenberg et al. 2008), whilst rates are not decreasing (Ohuma et al. 2023). As such, research into the psychological impact of either risk of or actual preterm birth is particularly important (Ionio et al. 2016; Worrall et al. 2024), given the long‐term adverse consequences to both mother and infant (Luu et al. 2017).

Women who have a preterm birth or who babies are admitted to NICU are therefore a population who require specific attention. It is well‐established that mothers of preterm infants experience anxiety to a higher degree comparative to mothers of term infants (Worrall et al. 2023). This may be in part exacerbated if the infant spends a period of time in the NICU (Garg et al. 2023). The NICU itself acts as a unique psychological stressor (Miles et al. 1993; Van Wyk et al. 2024), the effect of which can persist well into the first year after birth (Lean et al. 2018). In light of the increasing recognition of the value of postpartum‐specific tools (Meades and Ayers 2011), the PSAS (Fallon et al. 2016) and the PSAS‐RSF (Davies et al. 2021) have to date been used in two studies exploring anxiety in mothers of preterm infants (Worrall et al. 2023, 2024b). However, recent research has demonstrated that mothers of premature infants respond differently to items on the PSAS‐RSF than mothers of term infants (Worrall et al. 2024a), and so it requires modification for use in this specific population. Moreover, none of the items reflect the unique circumstances surrounding preterm birth, which may include infant NICU admission.

## 2 Methods

### 2.1 Aims

In this protocol we aimed to undertake the development and validation of the PSAS for use with mothers of premature infants and those with infants in the NICU as follows:

1. To adapt the existing PSAS‐RSF for use in mothers of premature infants (PSAS‐PTB).
2. To develop a scale for use with mothers of infants in the NICU (PSAS‐NICU) which could be used as a standalone scale for measuring anxiety and also as a ‘Velcro’ sub‐scale to be used in conjunction with any or all of the PSAS, PSAS‐RSF, PSAS‐RSF‐C, or the PSAS‐PTB.
3. To outline the process for validation of both the PSAS‐PTB and the PSAS‐NICU.

### 2.2 Overarching Study Design

The development of the PSAS‐PTB and PSAS‐NICU comprises of four main study stages, three of which concern the development of the scales (Stage 1: Patient and Public Involvement and Engagement Focus Groups; Stage 2: Expert Panel Survey; Stage 3: Cognitive Interviews), and one of which concerns the scale validation (Stage 4: Large‐scale On‐line Survey). Each of these will be discussed in turn, as they comprise of different analyses and methodologies. The development of the scales was iterative, meaning that each stage of the development informed the next stage of research.

### 2.3 The Research Team

The research team comprises multidisciplinary researchers, who for this specific development and validation, represent disciplinary backgrounds in psychology [S.W., P.C., S.A.S., V.F.], psychometric statistics [P.C.], implementation sciences [S.A.S., A.K.], obstetrics [A.K.], and women's health researchers [S.A.S., S.W., V.F., A.K.]. This collaborative approach to study design, data collection, and data analysis allowed for a holistic perspective to the scale development and cross‐disciplinary insight meant the final scale was comprehensive and grounded in the psychological and clinical evidence‐base.

## 3 Study Stages

### 3.1 Results of Study Stage 1: Patient and Public Involvement and Engagement Focus Groups

The initial stage of development comprised three Patient and Public Involvement and Engagement [PPIE] focus group discussions with stakeholders. At this stage, the research only intended to adapt the existing PSAS‐RSF (Davies et al. 2021) for use in mothers of premature infants to devise the PSAS‐PTB.

Participants for this stage were required to be over the age of 18 with a good understanding of the English language. These included professionals working in neonatal care or with mothers or premature infants, academics researching in the field, and parents of premature infants now aged between 0 and 12 months corrected age. Participants were recruited via volunteer sampling through posts on social media and/or via e‐mail through pre‐existing relationships within the research team. A total of 14 participants participated in one of three focus groups. They consisted of academics (*n* = 4), neonatal nurses (*n* = 2), mothers of premature infants (*n* = 3)—of which one mother had given birth to twins, clinical psychologists (*n* = 2), obstetricians (*n* = 2), a midwife (*n* = 1), and an obstetric physician (*n* = 1); however, some participants held dual roles. Two parents were removed as participants from the final analysis as they did not provide information beyond a brief background introduction about themselves, thus yielding a final sample of twelve participants.

Focus group discussions were held on‐line. Participants were shown a video detailing the work of the PSAS Global Collaborative to date (PSAS Global Collaborative 2023). Participants were then shown the PSAS‐RSF items by sub‐scale, and were asked to comment on the relevance and appropriateness of items for the specific population of mothers of premature infants. They were also invited to comment on the measure as a whole, including the length and scoring of the measure. Participants received a £20 voucher as reimbursement for their time. The focus groups lasted between 47 and 57 min (*M*_Time_ = 53 min). One of the research team led the focus groups [S.W.], which were facilitated by another member of the team [S.A.S.]. The focus groups were recorded and transcribed verbatim by one researcher [S.W.].

We analysed the transcripts of the focus group discussions using a summative content analysis (Hsieh and Shannon 2005), whereby data reflected the PSAS‐RSF sub‐scales and were discussed according to relevance of each item's ability to measure anxiety for mothers of premature infants, and the potential for the addition of new items. Changes to the potential items were based on the volume of responses. See Table 1 for a summary of the quotations.

**TABLE 1:** Summary of findings from the PPIE Groups.

Relevance	Potential additional items
Psychosocial adjustment to motherhood	
*‘And the I've worried I'm not going to get enough sleep. That's another one, because that's the one thing you know you're not with your baby, so you don't have all the night feeds to do. Obviously, you've got nighttime expressing and worries about your baby keeping you up.’* [neonatal nurse]	*‘Especially for parents going home if they have to kind of give additional medication or were going to go on to feed‐ feeding but like tube feeding in the community, lots of babies go‐ premature babies go home still tube feeding or on oxygen, and parents have to manage kind of anxieties around that.’* [clinical psychologist]
*‘…you know I‐ I have worried that I am not going to get enough sleep well, actually, if your baby's in NICU and being looked after, that's probably less of a worry whereas when you first go home and‐ and you know you've got a baby, that might be 2,3,4,5,6 weeks old, but that's your first night with that baby at home I think those are the, you know, they really lay awake with their eyes open most of those first nights, they are so‐ they are so on edge about it and they worry about it. So again, I think the timing is really important—’* [academic]	
*‘So the experience of a parent on a neonatal unit to a parent that then is at home is very different. For example, worried about finances when they're coming into the hospital, they've got to worry about parking which can cost quite a lot, meals, when they go home they don't necessarily then have those anxieties, or it could be that they do have outpatient appointment and that's [indiscernible]. But I just think you know is there a way of kind of altering the tool for parents in hospital to those that are outside of the hospital setting?’* [neonatal nurse]	*‘So to‐ to me I‐ I think that the big gap is the bonding. The baby's in a box or under a light, you know? And you know that physical interaction and the bonding and just the uncertainty. And also, if you just had a baby at 24 and 0, you know, what are the chances my baby's actually going to survive and how can I commit myself to this relationship when there is so much uncertainty about the survival and the quality of that survival for my newborn.’* [obstetrician]
*‘And then the finances again are interesting but I think, again, that's something when I've seen from a preterm perspective, they take their maternity leave early and then almost when their baby's ready to go home, their maternity leave has run out and then they'll be worried about their finances…’* [obstetrician]	
Practical infant care anxieties	
*‘I think the like milk intake and the weight are kind of obviously interrelated and obviously common probably across motherhood, but exasperated for, you know, premature babies often have the low birth weight…but I was like, less worried about the routine because I was just more concerned about trying to keep them alive, to be honest.’* [mother of a premature infant]	*‘I wonder if there needs to be like another sub part that's specific for like worries such as I'm worried about my baby being readmitted to the hospital, just based on what other people have been saying. I know it might be sensitive to include, but I'm worried that my baby's going to die’* [academic]
*‘So parents have kind of almost got that scaffold of routine sometimes to get into. So I wonder if that's maybe sometimes less of a‐ of a worry if they kind of have a neonatal experience.’* [clinical psychologist]	*‘But when you're in the thick of the NICU or you know neo admission I think it's more around you know how able parents feel to be involved in aspects of care. And yes, weight is important, but it might also be around parents' ability to be involved in feeding or it might be around their confidence in administering medication, it could be around confidence in changing babies nappy, you know it‐ those practical things feel quite different from the‐ the beginning of that experience to when you're ready to go home.’* [clinical psychologist]
*‘…if my baby was in the NICU, I would not be worried about any of these…because I would consider that all of this would be looked after by the neonatal team and actually I don't need to worry about the milk intake, I don't need to worry about the weight, I don't need to worry about them sleeping, I don't need to worry about a routine, I just want them to survive. And I think the difference in okay, here you go here's your baby, go home with some oxygen, and people saying to me I don't you know they're‐ they're‐ they're worried, not that they're going to sleep, that they're going to die overnight, that they need‐ like so I think the immediate worries in hospital are very different to the postnatal worries and they almost want to have these worries because that makes it normal‐‐’* [obstetrician]	*‘I think that some parents after discharge are worried about the oxygen therapy all these things that their infant maybe have to‐ to‐ to do at home and‐ and often they are very anxious how to manage all these things at home. So I think that maybe this part could be add[ed].’* [clinical psychologist]
*‘The only things I would say for a lot of premature infants, milk intake, and how they get milk actually, I think that needs to be more generalised about feeding because they might have a lot of anxiety about ongoing feeding, but actually we feed them via, you know, lines and via tube feed and they do get their milk intake but a parent might be very worried about what the long‐term journey to breastfeeding or bottle feeding may be, so…’* [neonatal nurse]	
Maternal competence and attachment anxieties	
*‘I don't like the word maternal competence because we might be making parents feel they have to prove their worth as a parent, and that is not the case for these babies, they just need the additional support to be able‐ to be able to parent within a neonatal setting, and sometimes beyond if they go home with extra care needs.’* [neonatal nurse]	*‘I don't know if this is also elsewhere in the questions, but I wonder if it would be worth having a question about whether people feel that the premature arrival of their baby was somehow their fault, because we do hear that a lot.’ ‘I feel so guilty.’ ‘My baby is here prematurely.’ ‘If I hadn't have done XYZ, then this wouldn't have happened.’* [neonatal nurse]
*‘You're responsible for the monitoring, you're responsible for knowing when something's not going properly, and you're responsible for getting someone's help that's required and‐ and I think that can be a difficult transition.’* [obstetrician]	*‘So I'd say it's [guilt] probably the main theme that comes up in my therapeutic work with parents. And I wonder if there's something about kind of like, I don't know how you would word this, but something about like I worry that my guilt is impacting my bonding with my baby, like, because the guilt, the guilt, can often be a barrier‐ or shame it can often move into shame‐ can often be a barrier to parents because they're so‐ they feel so guilty. And then because they then feel guilty that they have to leave their babies or have left their babies at some point.’* [clinical psychologist]
*‘…And I think negative thoughts about my relationship, but I think we want to kind of gauge where they are with how connected they feel and kind of capture both the negative and the more positive maybe so we can see how it's moving rather than just a negative thought at that particular time…?’* [neonatal nurse]	*‘…So maybe it could be useful to‐ to have an item in which parents could share the way they feel about the opportunity they have to bond when they are in NICU because I think that is very NICU dependent’* [clinical psychologist]
Infant safety and welfare anxieties	
*‘My one curiosity is whether that I worry that my baby will stop breathing while sleeping is actually maybe potentially quite triggering… just for some parents that‐ that is the reality of their experience’* [clinical psychologist]	*‘I think that one about potential readmission is…definitely one to consider because I think that's a huge factor for a lot of parents or‐ or I worry about my baby getting poorly again or‐ or something like that. You know, even getting a cold and things like that like parents are particularly heightened to any kind of typical bug that we‐ that you would be‐ that you may be worried about as a full‐term mum but not to the same extent.’* [clinical psychologist]
*‘I just wondered about the wording of the last one about worried about my baby being accidentally harmed by someone, and if you know the word accidentally was kind of essential to that and you know whether it was, I don't know. One of my anxieties when I was in hospital, probably because of the whole, like Lucy Letby thing was about like the people that were meant to actually be caring for the babies kind of doing something intentionally so, you know, not by accident.’* [mother of a premature infant]	*‘…One thing also about people being very hypervigilant about any future disabilities or brain damage or that kind of thing. Which again, completely understandable in the circumstances, especially when you've got an extreme proem that's had a very bumpy journey. It‐ it‐ it‐ it almost seems to haunt people like it's like a spectre…’* [neonatal nurse]
*‘But I'm‐ I mean, we're talking about severe morbidity, lifelong‐ you know, a totally altered life trajectory versus‐ and whether or not your baby's going to survive. I mean, it really is a completely different parenting, new parenting experience, I think, compared with most women who have a relatively physiological event on labour board and‐ or at home. So I yeah, I‐ I‐ I think they're‐ really there need to be those questions in there and also how is the mum dealing with what may have been a traumatic birth experience and how is that overlaying how they're coping with the neonatal course as well…’* [obstetrician/Academic]	*‘When we work with parents of very preterm baby they sometimes are worried about the‐ they think that their baby could die, and I think that this is also an‐ an important topic that go into their anxiety about their [indiscernible]. We they‐ as [Rose] said, they are worried that the baby could be have an impairment in the future, but also they are worried about the survival of their baby.’* [clinical psychologist]
*‘…There's something you know the‐ the dominant anxiety about a child's safety or welfare is probably based in the reality of the context that they're in, if they remain an impatient, which feels like you're measuring something very different than if you were at home with a baby at, you know 9 months or a year‐ I don't‐ it just feels like you're potentially measuring two different things—’* [clinical psychologist]	*‘Almost like that I worry about my baby's future.’* [clinical psychologist]
Potential additional items	
*‘I was just wondering if maybe like, preoccupation with baby's health or wellbeing could be a potential fear as well. So that sort of like ruminating, intrusive thoughts, that kind of thing.’* [academic]	
*‘I suppose that would depend on if you were gonna do a‐ so a measure for whilst in inpatient and then a measure for after because that would‐ it would essentially be two different parts of the journey.’* [clinical psychologist]	
*‘I was thinking maybe‐ maybe you need to produce one version for all‐ all parents, or all mothers of babies in the neonatal unit, which would have the same anxieties and not just preterm, but all babies that were admitted to the unit, and then maybe that could be used for the preterm and the non‐preterm babies in the special care unit and then‐ I mean I think this is a really important point.’* [midwife]	
*‘I was just going to say just thinking, if this is a tool that's already used across the board for sort of all mothers and infants, I think limiting it to just pre‐ premature infants, which is such a small number of what we get in a NICU, we get a lot of very sick term babies, and I don't want to be cutting those‐ that cohort out because actually that's our biggest cohort of‐ of families a lot of the time.’* [neonatal nurse]	
*‘Yeah, it's in terms of a bit of a gap, it's about aspiration. I mean, when we're parents, we have a new baby we're, you know, we have this vision of who that baby may become. You're an independent, physically, and mentally capable person who will contribute to society and be happy. And probably the happiness is the most important thing I should have probably said that first, but that probably speaks to my academic mindset. But I‐ and there's nothing in here about that and when you're dealing with the baby in the nursery, who may be having seizures, who may have retinopathy, who there‐ or may have had the bowel removed because of necrotising enterocollitis, whatever it is, your‐ your aspirations become diminished. Or certainly you have uncertainty about how much you can hope for your‐ for your new baby..’* [obstetrician/Academic]	
Length, scoring, and time period	
*‘…my experience of, you know, thinking in the first year of life and premature parents, their general resource is low and their ability to reflect and be aware and their self‐awareness isn't as good as it may previously be, and I wonder if kind of a shorter measure, may be more helpful.’* [clinical psychologist]	
*‘I think I agree, and there's a lot of others you know the EPDS is 7 days. There's a lot of other anxiety questionnaires. It's in the last 7 days, so that goes along with a lot of other validated measures out there that are used in clinic‐ in clinical and research I think, and also I think once you get beyond that, I think there's issues around recall bias. So I would think 7 days is perfectly reasonable.’* [academic]	
*‘You know, I tend to think that anything up to sort of 25 to 30 items is fine, especially if it's quite simple like this. And not, you know, not very long rigmarole reading about it.’* [academic]	

Whilst participants vocalised the value of a perinatal‐specific tool, clear avenues for the adaptation of the PSAS‐RSF were prevalent. Most notably, stakeholders agreed one overarching preterm‐specific tool would not capture the unique experience of having an infant admitted to the NICU in the absence of prematurity, and that restricting the scope of the psychometric development to just one preterm‐specific tool would limit the scope, usability, and implementation of any such tool. It was therefore agreed with the wider research team that the remaining stages of development would focus on both a preterm‐specific (PSAS‐PTB) and a NICU‐specific (PSAS‐NICU) adaptation of the PSAS. Items for the initial versions of the PSAS‐PTB and the PSAS‐NICU were based upon the analysis of the PPIE groups, review of the literature base, and consultation with the wider research team [S.W., V.F., P.C., A.K., S.A.S.]; and these were then implemented into Study Stage 2.

### 3.2 Results of Study Stage 2: Expert Panel Survey

Following the PPIE focus group discussions and the initial development of items for both scales, these items were subsequently rated by a group of experts in the field via an on‐line survey to establish the comprehensibility and understanding of the scales as a whole and the individual items.

Participants were again required to be over the age of 18 with a good understanding of the English language and had to have expertise in either psychometric scale development, perinatal psychology, maternal anxiety, or preterm birth. Participants were recruited via e‐mails from the research team to their extended collaborative networks and also to those who took part in the PPIE focus groups, as part of consented re‐contact. A total of 16 participants participated in the on‐line survey. They consisted of psychologists (*n* = 6), clinical psychologists (*n* = 2), psychiatrists (*n* = 1), midwives (*n* = 3), nurses (*n* = 1), obstetricians (*n* = 1), and researchers (*n* = 7); however, some participants held dual roles.

Participants responded to the survey which was hosted on Qualtrics and were presented with the PSAS‐RSF and the proposed PSAS‐PTB and PSAS‐NICU items. Participants were presented with each item in‐turn and were asked to rate the relevance of the item in measuring postpartum anxiety in mothers of premature infants on a scale from 1 to 4 (1 = Not at all relevant, 2 = Somewhat relevant, 3 = Quite relevant, 4 = Highly relevant), before being invited to provide any additional feedback in relation to the items. For each scale, they were asked to identify which items they would definitely retain, definitely remove, or modify (with reasons for modification), before being asked to suggest any additional items (if any). Finally, they were also asked general questions about the scales including ideal length, consideration of functioning as well as frequency of anxiety, time periods in which to use such a tool, and scoring of the scales. The survey took approximately 15 min to complete.

Quantitative data were analysed descriptively. Qualitative data via open textboxes were brief, functional, and informative and so were not subjected to a specific analytical methodology. Decisions on retention, removal, and modification of items as suggested by the expert panel, were then discussed with the research team [S.W., P.C., A.K., S.A.S., V.F.].

Generally, proposed items for the PSAS‐PTB and PSAS‐NICU were rated highly in terms of their relevance, and comments on the items were positive. Relevance of items across the proposed PSAS‐PTB and PSAS‐NICU can be found in Tables 2 and 3, respectively; whilst details of which items were identified for retention, removal, or modification, with associated comments can be found in Supporting Information S1: Tables 1–2. As it was intended that the PSAS‐NICU was to be used as both a standalone scale and as an additional ‘Velcro’ to the other derivatives of the PSAS, it was important that items did not overlap with those already addressed in the PSAS‐PTB. Therefore, items on both the proposed PSAS‐PTB and PSAS‐NICU were scrutinized by the research team [S.W., S.A.S., V.F., A.K., P.C.] and items were moved between the two scales in accordance with where they were most appropriate and pertinent. Additionally, for the PSAS‐NICU, there was a need to ensure the items could be asked both whilst on the NICU and when infants had been discharged from the NICU. As such, *‘When my baby was in NICU, I am/have felt…’* was added before the items on the PSAS‐NICU only.

**TABLE 2:** PSAS‐PTB relevance for use in mothers of premature infants (rated from 1 = not at all relevant, 4 = highly relevant), with comments (*N* = 16).

Item	Mean (SD)	Comments
I have worried I cannot provide the right care for my premature baby	3.56 (0.63)	*Well worded*
		*depends on needs of preterm infant*
		*may be a normal reaction felt by most mothers of a proem baby*
		*I think that feeling not knowing how to behave with a baby who might have additional needs might be relevant for parents of premature infants*
I have felt that my baby being born prematurely was my fault	3.50 (0.73)	*High levels of guilt and felt sense of responsibility*
		*Feeling responsabile [sic] for not keeping the infant inside long enough might be common fear for mothers of premature infants*
		*I added too!*
		*I think this is very common, all mothers with an adverse outcome have some degree of self blame. As it is universal it is unlikely to be a strong discriminator between those who have some and those who have severe anxiety.*
I have worried about my baby becoming ill because of their prematurity (*N* = 15)	3.53 (0.64)	*Premature infants are more likely to become unwell and parents are reminded of this regularly*
		*Would expect most mothers of proem babies may feel this*
		*Feeling anxious about the health of an infant perceived as particularly vulnerable might be common for mothers of premature infants*
		*The NICU environment can cause this*
I have worried about my baby's future development because of their prematurity	3.63 (0.62)	*Mentioned throughout Neonatal experience and inpatient stay. Can be a big concern due to the uncertainty and waiting to see if baby's reach particular milestones*
		*Reasonable to worry about this*
		*Feeling anxious about the health of an infant perceived as particularly vulnerable might be common for mothers of premature infants*
		*Less immediate than current illness, so less relevant.*
I have worried about my baby's future quality of life because of their prematurity	3.44 (0.63)	*As above*
		*Reasonable to worry about this*
		*This fear might be common for mothers of premature infants as they might not know what are the consequences of being born prematurely, but also other mothers might be worried about their baby's development*
I have not been able to stop thinking about the size of my baby	3.31 (0.70)	*Consider removing. This is basically the same as item 9.*
		*Not relevant to all families*
		*Recurrent negative thoughts about the health of an infant perceived as particularly vulnerable might be common for mothers of premature infants*
I have worried about my future quality of life because of my baby's prematurity	3.00 (0.82)	*Reasonable worry*
		*This fear might be common for mothers of premature infants as they might not know what are the consequences of being born prematurely, but also other mothers might be worried about their baby's development*
		*I think parents would prioritise their baby's quality of life rather than their quality of life.*
I have worried that my baby is not gaining enough weight	3.31 (0.70)	*Feeling anxious about the health of an infant perceived as particularly vulnerable might be common for mothers of premature infants*
I have worried about the size of my baby	3.06 (0.77)	*Very similar to item 6. I actually prefer this item to item 6, consider removing.*
		*Feeling anxious about the growth of an infant perceived as particularly vulnerable as born smaller than expected might be common for mothers of premature infants*
		*Though very similar to Q6, I don't think both questions would be needed in a clinical scale.*
I have worried that I will do further harm to my infant if I interact with them because of their prematurity	3.06 (0.93)	*Most relevant within the Neonatal setting as parents do not want to touch their babies as they appear fragile and delicate*
		*Not so reasonable*
		*In some ways mothers of premature infants might feel they ‘caused’ the prematurity, but the item seem a bit convoluted*
I have worried about my infant's appearance	2.63 (0.81)	*'due to their prematurity' to make sure that it is consistent with the other items in the measure and that it is proem‐specific.*
		*It might be a bit relevant if as ‘appearance’ they mean being too little/short*
I have felt uncertain about my baby's future	3.25 (0.86)	*Already captured in the developmental, appearance, and quality of life items about the infant's future. This item in and of itself is not proem‐specific.*
		*This could be common for many parents*

**TABLE 3:** PSAS‐NICU relevance for use in mothers with infants admitted to NICU (rated from 1 = not at all relevant, 4 = highly relevant), with comments (*N* = 16).

Item	Mean (SD)	Comments
I have worried about providing additional medical care for my baby.	3.25 (0.86)	*Related to worries about health*
I have worried about my baby being re‐admitted to hospital.	3.56 (0.89)	*Normal*
		*Related to worries about health*
I have worried about the survival of my infant.	3.75 (0.78)	*‘my baby’ not ‘my infant’ so it aligns with the other items in the scale.*
		*Normal*
		*After a shocking experience in which mum could have lost the baby this is an understandable fear*
I have worried about medical equipment on or near my baby.	3.19 (0.54)	*Related to not know about medical care for premature infants*
I have worried about how much time I have been able to spend with my baby.	3.50 (0.63)	*…since they have been admitted to NICU.*
		*depends on circumstances*
		*Related to fear of loosing baby*
		*The guilt of not having the normal initial postnatal period to bond etc.*
I have worried about my baby becoming exposed to infection in hospital settings.	3.44 (0.63)	*This could be common for parents but particularly relevant for infants perceived as vulnerable*
I have not been able to stop thinking about how ill my baby is.	3.38 (0.89)	*……since they have been admitted to NICU.*
		*depends on reality of the situation*
		*Related to Fear of health problems*
I have repeatedly worried about changes to my baby's condition.	3.69 (0.79)	*…since they have been admitted to NICU.*
		*Related to not feeling in control and that infant might be vulnerable again*
I have repeatedly asked about the status of my baby's health.	3.38 (0.89)	*…since they have been admitted to NICU.*
		*Depends on situation*
		*This could be common for many parents with children in hospital*
I have worried that interacting with my baby will interfere with their medical care.	3.25 (0.86)	*…since they have been admitted to NICU.*
		*Related to not knowing how to care for a premature/vulnerable infant*
I have worried about developing a relationship with my baby because I am concerned about their survival.	3.69 (0.60)	*…since they have been admitted to NICU.*
		*Understandable*
		*Fear of attach to someone who might ‘leave’*
I have felt frightened in the NICU environment.	3.38 (0.72)	*Not very specific*
		*It is awful environment. Can sit with you. The sounds. The other babies.*
I have worried that my baby is in pain.	3.50 (0.82)	*…since they have been admitted to NICU.*
		*can be a reasonable worry*
		*Related to not knowing how the care for a premature/vulnerable infant works*
I have worried that I do not get alone time with my baby.	3.06 (0.85)	*…since they have been admitted to NICU.*
		*depends on circumstances*
		*It depends on how the NICU is organised*
I have felt unable to protect my baby from harm.	3.63 (0.62)	*…since they have been admitted to NICU.*
		*Fear of not be “good enough” or to have caused the premature birth*

Participants also provided general comments including ideal length, consideration of functioning as well as frequency of anxiety, time periods in which to use such a tool, and scoring of the scales for both the PSAS generally (Supporting Information S1: Table 3). Whilst some items on the PSAS‐RSF were rated highly for relevance and were commented upon with respect to potential items to include (see Supporting Information S1: Tables 4 and 5) only the proposed new items for the PSAS‐PTB and PSAS‐NICU were rated in Study Stage 3—Cognitive Interviews.

### 3.3 Results of Study Stage 3: Cognitive Interviews

Following the results of the expert panel survey, the final stage of development consisted of cognitive interviews, which is a qualitative research approach which aims to assess and improve the validity of surveys (Scott et al. 2021). Cognitive interviews enable the researcher to understand how participants are interpreting the meaning of questions, including understanding of terminology, errors in the wording of questions, or potential issues with response options.

Both newly proposed scales were subjected to cognitive interviews with mothers of preterm infants (*n* = 1), mothers whose infants had been admitted to NICU (*n* = 2), and/or mothers who had experienced both preterm birth and their infants had been admitted to NICU (*n* = 2). Mothers were either academic researchers (*n* = 4) or entrepreneurs (*n* = 1), with a mean age of 36 years (SD = 1.41), and had given birth at an average of 36^+1‐2^ weeks' gestation. Their infants' mean age at time of interview was 19 months (SD = 10.05) and the average NICU stay for those who had an infant admitted (*n* = 4) was 14.00 days (SD = 6.98). One mother had given birth to twins. Participants were recruited in the same manner as the PPIE groups and those who made contact were e‐mailed an information sheet and consent form; which once completed, led to an invitation to the interview. Participants were required to be over the age of 18, and have a good understanding of the English language. Participants had to have had a live infant either born prematurely (< 37 weeks') aged between birth and 5 years corrected age with or without NICU admission; or had a live infant born at term aged between birth and 5 years who had been admitted to the NICU, in the absence of prematurity. Participants received a £20 voucher as reimbursement for their time.

Cognitive interviews are typically conducted with small numbers of participants (Willis 2005). As such, five cognitive interviews were conducted by one researcher who had received formal training in the methodology [S.W.]. Cognitive interviews lasted between 47 and 121 min (*M*_Time_ = 73.53), and were conducted, recorded, and auto‐transcribed on Microsoft Teams. During the interview, participants were trained in the think‐aloud technique, asked some demographic questions, given an overview of the scales, before being shown either the PSAS‐PTB, or PSAS‐NICU, or both, dependent upon their circumstances. Participants were told to be as honest as possible in their interpretation of the questions, and that the interviewer would not be upset or offended by any of their answers, as outlined in guidance for cognitive interviews (Willis 2005). Using the think‐aloud technique, allowed participants to be asked to provide an account of what they were thinking whilst responding to the item, and probing techniques consisting of pointed questions about participant's thinking to target key areas which may be problematic (e.g., surrounding comprehension, hesitation). Common probes included: ‘*What are you thinking when you see X term?’;‘ Do the response options allow you to adequately respond to the question?’.* Probes were asked concurrently, after the participant had reviewed each individual item. Participants responded to each item in turn, before being asked some general questions about the scale such as relevance, length, and scoring.

Interviews transcripts were checked for accuracy of recording [S.W.], and upon completion of the cognitive interviews, the whole research team [S.W., P.C., A.K., S.A.S., V.F.] met and discussed participants responses to each item, to identify both dominant issues (i.e., particular items with repeated issues) and discoveries (i.e., an issue only emerging in a single interview, but may still affect item responses) in‐line with current and contemporary guidance on cognitive interviews (Balza et al. 2022; Willis 2015). Adaptations to items were decided upon and agreed by the group, iteratively and by consensus.

Participant responses to items and how they changed can be seen in Tables 4 and 5. Response options and the 7‐day recall period were largely deemed appropriate for both scales.

**TABLE 4:** Iterations of the PSAS‐PTB following the cognitive interviews, with participant responses.

	Original item	Participant responses	Final item
1.	I have worried I cannot provide the right care for my premature baby.	*‘The word worried is quite an accessible term’—*Participant 1	I have worried I cannot provide the right care for my premature baby.
		*‘My kind of instinct was to answer it as in kind of doing all the right things to keep the baby safe and alive’*—Participant 1	
		*‘I think it's* [the right care] *kind of quite vague’*—Participant 1	
		*‘I was thinking about kind of how it just wasn't even on my radar, but it could be a thing and also that premature babies need a different care. So I think, yeah, I'm thinking about kind of how just unprepared I was and how yeah‐ and how I just didn't know how to start.’*—Participant 3	
		*‘When I found out she was coming early and then also this first few months of, oh, my God, what do I do?’*—Participant 3	
		*‘[I'm thinking] in terms of medication, feeding tube, timed feeds, top up feeds all the things that‐ it just was not on my radar until she was born… yeah, just not having any awareness of how you deal with all those things.’*—Participant 3	
		*‘I wasn't expecting her to come, and then she suddenly was there and absolutely tiny and I had a tube fed baby and had no idea what I was doing and first time mom and everything so.’*—Participant 4	
		*‘That initially was the right at the start when she was tube fed, because that was the biggest thing that was new, even though obviously you have to change nappies and do all that‐ that's everyone does that, don't they?’*—Participant 4	
2.	I have felt that my baby being born prematurely was my fault.	*‘I kind of think seeing that written down like it is could potentially be a bit triggering’*—Participant 1	I have felt that my baby being born prematurely was my fault.
		*‘I think that question is really clear…like I don't think there is any ambiguity in it’*—Participant 1	
		*‘I think the only way to kind of really interpret that is you know did I‐ did I do something that kind of made the baby be born early’*—Participant 1	
		‘[Hesitates with response options] *I don't feel like they fitted quite as well with this one, but I‐ I understand there has to be like a kind of uniform option for like completing this.’*—Participant 1	
		*‘There's so much guilt, even though, like I know I have been categorically told that medically it was not my fault.’*—Participant 3	
		*‘I'm thinking about… I would‐ used to think back over my pregnancy because I was ill during my pregnancy, did that cause it? Was it something I did? And how I‐ did I make the right choices in the days leading up to her birth?’*—Participant 3	
		*‘So my initial thought was I know that her being born premature it wasn't my fault and you know, I'm fit and healthy… that would be the type of thing I would blame on it because I don't drink when I'm pregnant or, you know, that kind of thing. So that's what goes through my head initially anyway.’*—Participant 4	
3.	I have worried about my baby becoming unwell because of their prematurity.	*‘I immediately think about my littlest who had like respiratory issues… kind of like possible risk of infection and worried about visitor and people not sanitising, washing their hands and things.’*—Participant 1	I have worried about my baby becoming unwell because of their prematurity.
		‘[Unwell interpreted as] *Bugs, illnesses, infection’*—Participant 1	
		*‘So yeah, so I kind of, I just feel like it's constantly‐‐ I'm thinking about how like I am on high alert, like if somebody has a cold or a cough, I don't really want them near her.’*—Participant 3	
		*‘I'm thinking in terms of mostly respiratory stuff because that was some of her‐ that's some of her risks. And so kind of chest infections and so on. So I'm kind of thinking probably more kind of like illness she can catch off other people rather than things that develop internally.’*—Participant 3	
		*‘…but I definitely was worried and especially when she was tiny. So I would say often for that one because when she was tiny, they're so small and you just, it wasn't like we didn't want people to hold her…I was worried that she, you know, if she got a cold’*—Participant 4	
4.	I have worried about my baby's future development because of their prematurity.	*‘The first thing that comes to mind is like the corrected age or adjusted age… I think my answer to this would kind of be dependent on how close they were to that like 1 year mark’*—Participant 1	I have worried about my baby's future development because of their prematurity.
		*‘Just thinking about development is like a broad thing… like physical development, like eating milestones, cognitive… my mind went more to kind of like meeting milestones in general.’*—Participant 1	
		*‘… I'm thinking about how know you shouldn't but you do compare to other babies and seeing that she's not hit them’*—Participant 3	
		*‘I'm thinking about kind of like the milestones and how the stuff about like the apps, the Wonder Years app, but I stopped using it because we weren't hitting any of them and it was just too upsetting and kind of it sort of stuff doesn't apply and kind of you just don't know what sort of timeline you're on’*—Participant 3	
		*‘I'm thinking sort of developmental milestones, so mostly cognitive, but also motor and kind of thinking about in terms of‐ if, if she's kind of smiling, clapping, laughing on‐ on schedule…’* ‐Participant 3	
		*‘So I'm thinking about it like, okay, if we're not doing this now, does it mean that other stuff's going to be delayed? And what does it mean for her in terms of sort of school social development? Making her way through the world and stuff.’*—Participant 3	
		*‘…she was so small and she like I say, she didn't‐ she didn't smile till she was 12 weeks…. And I remember having the cards that like 1 week old and whatever…and I thought, do I do these now?’*—Participant 4	
		*‘I mean you do think about the walking and the talking…’*—Participant 4	
5.	I have worried about my baby's future quality of life because of their prematurity.	*‘It isn't something that crossed my mind… when I read the question then I'm like should I have been kind of thinking about that more?’*—Participant 1	I have worried about my baby's future quality of life because of their prematurity.
		[Asked about QoL] *‘Quite broad in terms of like if they'll be able to do all the things that I kind of have growing up, like have friendships, have relationships, be in kind of employment, go to school, college, University… be happy.’*—Participant 1	
		*‘I was thinking about in terms of I think about if she is going to struggle at school, if she's going to be more poorly with illnesses like coughs, colds, bugs, because she's more vulnerable. She's also very small, even for even now. So is she going to‐ is she going to struggle to keep up when she's playing with people? So yeah, I'm thinking about kind of how things that have happened or that she has because of being small, is that‐ how is that going to impact her further on?’*—Participant 3	
		*‘I think development I'm‐ I'm thinking milestones to be honest with you. Quality life, I'm thinking making sure she's happy.’*—Participant 3	
		*‘So I think of that in terms of like sort of her social development and health and whether she'll be okay, will she need extra support when she's older? Will she have any sort of learning disabilities, that kind of thing is what I think of that.’*—Participant 4	
6.	I have worried about my future quality of life because of my baby's prematurity.	*‘All of the same things* [as above] *but also with the added element of self‐care’*—Participant 1	I have worried about my future quality of life because of my baby's prematurity.
		*‘I think‐ I think I'm kind of thinking about how‐ my husband and I, we've just been thrown into this. This is now life and we just deal with it and make sure she's okay. I've never actually‐ I've realised I've never actually thought about impact on me or considered that it would do.’*—Participant 3	
		*‘I wouldn't have to quit work and my when I think of quality of life, it's like, what am I going to have to give up? So do I have to quit work? You know, would we‐ this stop us having another child, that kind of thing.’*—Participant 4	
7.	I have worried that my baby is not gaining enough weight because of their prematurity.	*‘I quite like the fact that it says enough weight rather than just weight because obviously they're like charting it and looking at the trajectory and things’*—Participant 1	I have worried that my baby is not gaining enough weight because of their prematurity.
		*‘You know when it says “I've worried that my baby is not gaining enough weight because of their prematurity”… I don't know whether my concerns are necessarily because of their prematurity… they are just because of the small size which I know they come hand‐in‐hand’*—Participant 1	
		*‘So I'm thinking back over kind of the weight gain struggles we've had and the precautions I used to have to take and still do have to take around this and how yeah, I had to have her weighed a lot more regularly than a non‐premature baby.’*—Participant 3	
		*‘…that‐ that weight gain, it's just it's inherent basically also thinking about other like NICU and preemie mums that we know like we've got a group of friends from the neonatal unit. Now I'm thinking about how like, yeah, like weight gain is a massive thing and we all celebrate each other's weight gains as well for the babies.’*—Participant 3	
		*‘So I think it's hard to differentiate between gaining enough and rate of gain. Gaining enough weight definitely makes sense, because there's kind of limits without like making sure you regain your birth weight and so and however much more to be able to leave NICU and gaining enough weight to stay tracking whatever centile you're on. But one thing in our experience that we found was it was the rate of weight gain as well. If that's lowered, it started ringing alarm bells.’*—Participant 3	
		*‘…we got told often she's not gaining enough weight. So this is a phrase that got used a lot. So I'd say this is a very common phrase that people with babies who aren't gaining enough weight…because often you start off and they weigh them, don't they? And then they're on their chart and it's if they kind of deviate too much.’*—Participant 4	
8.	I have worried about the small size of my baby.	*‘…because the previous question was focussed on weight, then I would assume that this one was asking for things other than weight. So like, you know, length.’*—Participant 1	I have worried about the small size of my baby.
		*‘I'm just thinking about how tiny she was at the start and how fragile and how it just kind of compounds your feelings of how vulnerable they are.’*—Participant 3	
		*‘I'm thinking about kind of‐ also kind of what that means is in how small she is. Is that going to affect her developmentally because she's smaller?’*—Participant 3	
		*‘I'm thinking about kind of I would‐ I would like worry for her and her physical health, but also the worries of what are we going to run into and what does it mean for her going out in the world? Are people going to judge her in terms of size?’*—Participant 3	
		*‘I was thinking about like the physical size of her in my hands and the weights.’*—Participant 3	
		*‘That's what comes into my head. Cus obviously we've done the weight, kind of weight is tied in with length but by size I imagine how big was she, oh she was about this big, so that's what I think of…they're obviously linked, but yeah, it's kind of you think I think length for that one for size, yeah*.’—Participant 4	
9.	I have worried that I will do further harm to my infant if I interact with them because of their prematurity.	*‘I don't like the word further harm because it implies that I've already caused some harm or that they've come to harm like some other way.’*—Participant 1	I have worried that I will harm my baby if I interact with them because of their prematurity.
		*‘I notice that they've said infant on this one rather than baby, which baby is [a] like much more kind of familiar term.’*—Participant 1	
		*‘…like picking them up, getting them dressed, putting them in the care seat, like having them in the car seat in general… you know, kind of like trying to play with them, like being a bit more gentle compared to with my little girl* [who was not born prematurely].’—Participant 1	
		*‘But by harm I straight away think about kind of like accidental harm.’*—Participant 3	
		*‘I think it implies that the mother's already done something, which, considering the question about fault and guilt I think might be a little bit tricky… further harm implies that I've already done some harm.’*—Participant 3	
		‘I'm thinking of picking her up, holding her…but then I'm thinking of playing on the floor with her when…she was a bit older.’—Participant 3	
		*‘So I read that as like a social interaction. So like face‐ talking to her, like eye contact, that kind of thing like cuddling and holding as well but more of the looking at her face and letting her see our faces and just, you know, soak up the world in that way. That's what it makes me think.’*—Participant 4	
10.	I have worried about the low birthweight of my baby.	*‘I think my first thought is like should that be kind of grouped with the question about weight or size and like why it's kind of separated out?’*—Participant 1	I have worried about the low birthweight of my baby.
		*‘There's already been a question about like gaining enough weight, then I kind of think like, oh this one's kind of specific to obviously birthweight.’*—Participant 1	
		*‘So yeah, straight away thinking back to the stuff that I talked‐ we talked about when I was saying about weight gain and small size kind of thing, worrying about what it means. Thinking about how a low birth weight can impact development, but also all the horror stories and scare stories you hear about low birth weights being risk for developmental delays.’*—Participant 3	
		*‘Now, because you've already asked that I'm now thinking, okay, no I'm thinking about her actual birth weight at the time….separate to what happened to her weight after she was born.’*—Participant 4	
Response options		*‘I couldn't really think of anything more appropriate that would kind of cover the questions as well.’*—Participant 1	
		*‘Not at all, obviously like haven't thought about it. Not very often like it might have just cropped into my mind like once or twice. Often would kind of be like, maybe like once, once a day, then almost always I would interpret as something that's kind of like constantly playing on my mind’.*—Participant 1	
		*‘I don't know if there needs to be a distinction as in like kind of I used to but not so much now or if that's just complicating things a bit more.’*—Participant 3	
		*‘…or even if you were to say to people that answer these about the first 6 months or something rather than up to 12.’*—Participant 3	
		*‘I wonder if it's worth putting a sometimes in the middle, but then does that give you anything? Maybe not.’*—Participant 4	
Comments on the scale		*‘It's‐ it's neither too long or too short…it feels quite comfortable to do.’*—Participant 3	
		*‘You probably could ask another couple of questions. I don't think people would be, you know, because it's quite a short scale, but I think it actually covers quite a lot of things in there.’*—Participant 4	
		*‘I mean, because you don't ask about feeding specifically, do you and whether it's worth asking something about that, because I definitely think…that was definitely a big anxiety.’*—Participant 4	
		*‘The sleep thing. I remember getting really worried…I think sleep and the feeding, they were big factors.’*—Participant 4	

**TABLE 5:** Iterations of the PSAS‐NICU following the cognitive interviews, with participant responses.

Item		Participant responses	Final item
When my baby was in NICU, I am/have felt…			
1.	Worried about having to provide additional medical care for them.	*‘And trying to under‐ yeah, understand the question in terms of additional medical care, is that once I take them home, when it becomes my responsibility or is that when they're in NICU?’*—Participant 2	I have worried about providing additional medical care for my baby.
		*‘I'm thinking about kind of how even though the doctors and the nurses are there, there is stuff that you need to be doing because it's stretched in the NHS, they can't be there every second of every day.’*—Participant 3	
		*‘I'm thinking about the oh, my God, the oxygen, the bloody oxygen monitor that used to beep every 10 s and alarm all the time… yeah, it was just like it was something else to be focusing on as well as, oh my God, I've just given birth’*—Participant 3	
		*‘…I'm thinking about tubes, equipment, that kind of thing.’*—Participant 3	
		*‘I'm thinking about the things that we have to specifically do like my husband had to specifically do the feed the tube feed I had‐ I was specifically reattaching the oxygen monitor’*—Participant 3	
		*‘I'd often worry about providing additional medical care for mostly it was the tube feeding because we were like, oh, I don't know what to do, but we wanted to go home.’*—Participant 4	
		*‘Whether any of the complications or conditions that he had would then cause further issue and possibly would it be a case of needing further medical advice on that…’*—Participant 5	
		*‘…it would probably be on the unit, a case of what is that going to entail? But outside of hospital, more geared towards, could there be other things that are then needed further down the line as he gets older?’*—Participant 5	
2.	Concerned about them being readmitted to hospital in the future.	*‘I think that's really clear.’*—Participant 2	I have felt concerned about my baby being readmitted to hospital in the future.
		*‘I'm kind of thinking about kind of what it would mean for her and risks it would‐ additional risks for her if we'd have had to go back in.’*—Participant 3	
		*‘… But you felt like every time the health visitor or the tube feed team came around as lovely as they were, that you're always going to have to go back… but I just felt like I was being constantly monitored as well.’*—Participant 4	
		*‘I think instinctively as a mum in that position you automatically just think, yeah.’*—Participant 5	
3.	Frightened about their survival.	*‘Frightened, I think is totally appropriate. And survival. Yeah. I mean, I think it's probably better to focus on survival than death.’*—Participant 2	I have felt frightened about my baby's survival.
		*‘…kind of like how severe the medical needs can be or like how poorly some of the babies could be.’*—Participant 3	
		*‘I don't think that goes away to some extent, but definitely when she was really little it was. And I said in NICU, is she going to live?’*—Participant 4	
		*‘…and to me the concern for their survival is‐ is the initial fear.’*—Participant 5	
4.	Concerned about them needing support from medical equipment.	*‘I'm thinking about kind of oxygen and medication and IVs and like the you know oxygen saturation the monitors’*—Participant 2	I have felt concerned about my baby needing continuous support from medical equipment.
		*‘I guess my only thing that I think would be helpful with something like that would maybe be an example… I know it must be difficult because you don't want to lead people too much’*—Participant 2	
		*‘I'm thinking about [name of child] being on the feeding tube for so long and the oxygen when she was first born and the heated cot. So I'm thinking about all the different things that she needed so and then I was concerned about if that meant that we'd be there for a really long time’*—Participant 3	
		*‘… the medical equipment and thinking about all the tubes and wires and lights and stuff kind of all the physical bulky stuff as well. I'm thinking about the incubator as well.’*—Participant 3	
		*‘So I guess with that you could sort of say, needing support or continuing support if you're getting people to think about after that because yeah, I would say we knew she was only going to be on the tube for a short time.’*—Participant 4	
		*‘Medical equipment seems more serious, I think so medical care might be you might have to go back to the doctors more you might need, you know, support from you know, different services like health visitors or can't think what they're called now, the team, the‐ the feeding team. But then medical equipment seems a lot more like, you know, might need oxygen, might need actual like solid things.’*—Participant 4	
		*‘Would it be a case of, and me worrying that outside equipment other than what we're‐ we're already dealing with is needed.’*—Participant 5	
		*‘…if it was further down the line, I would probably then be thinking is it more concern of his like development or you know that type of medical equipment because I'd be thinking, well, he'll be home, but is there, is there going to be implications of his stay kind of thing?’*—Participant 5	
5.	Worried about how much time I have been able to spend with them.	*‘I guess that the only thing that I would be looking at that question and trying to respond to it appropriately was like ‘have been able to’. So it's not like are you asking me how much I wanted to or you asking me how much I‐ I could in terms of my physical health or in terms of like all the rules in NICU… just thinking it might be all of them you know in one question.’*—Participant 2	I have worried about how much time I have been able to spend with my baby.
		*‘I didn't leave. So I'm thinking about how I was fortunate that I could‐ I just moved in.’*—Participant 3	
		*‘…so have jumped out at me. It's like I don't know why. Just like oh, like I am able to spend with them I would think because obviously they're present they're with you, is what that makes me think so yeah… I think it just makes you seem like it's in the past, and I think if you've got, you are worried your baby's going to die, especially if they're a premature baby and you might get very‐ it's like you're talking about the past rather than the present… I think it just made me think, well, that's making me think about the past by and if I'm in NICU filling this in, I am with my baby.’*—Participant 4	
		*‘So for how much time I've been able to spend with them is initially, the thought goes to skin to skin contact and how little opportunity there is for that when you've got a baby in the incubator, and wires coming off them. Then a case of is me spending time with him me just being in the room, or is me spending time with him me holding him, you know me doing what I would say a‐ a new‐ a new mum should be doing.’*—Participant 5	
		*‘Because I'd‐ I'd feel like if you asked me if I was worried about how much time I've been able to spend with him if it was based on the last 7 days and I'd been discharged I'd imagine I'd have felt like I'd spent enough time with him, but I think you‐ your brain instantly goes back to the beginning and when it sort of felt like it got robbed off you.’*—Participant 5	
6.	Frightened about my baby becoming exposed to infection.	*‘You think of like hospital acquired infections… but you can't really say my baby becoming sick because… I think you're specifically asking questions around the infections there.’*—Participant 2	I have felt frightened about my baby becoming exposed to infection.
		*‘I think exposed to infection feels too… scientific… I can't think of a better word because that is very specific isn't it? …Maybe coming in contact with.’*—Participant 2	
		*‘I immediately thinking about how I was concerned about people from the outside bringing things in because we were in a little a little sterile bubble.’*—Participant 3	
		*‘I'm thinking more about that‐ that people coming in and bringing in illnesses with them rather than something in the hospital.’*—Participant 3	
		*‘I'm thinking respiratory again because that was actually‐ and respiratory and stomach bug, like gastro, cus those were [name of child's] biggest vulnerabilities.’*—Participant 3	
		*‘It makes me think of, yeah, sort of bugs like, you know, sickness bugs, diarrhoea, COVID, those kind of colds, coughs. That's what it makes me think of, not something more, you know, HIV or anything.’*—Participant 4	
		*‘Initially, like viruses, coughs, colds, anything that would make him really vulnerable to me.’*—Participant 5	
7.	Not been able to stop thinking about how unwell they are.	*‘[Hesitates] I would put that as not able to stop thinking about how unwell they are… I would probably use were. I would read that as… I felt not able to stop thinking about how unwell they were.’*—Participant 2	I have not been able to stop thinking about how unwell my baby is.
		*‘That one didn't apply to me as much because I didn't try to stop. I think it was just obviously, in such a bubble I didn't think of anything else or try to stop because that was all I could focus on.’*—Participant 3	
		*‘I just‐ I didn't think about anything else except [name of child]. I think if I'd have been going home and coming back and going to work and trying to do normal life, then that would have applied more… not because I wasn't worried, but because I did nothing but worry.’*—Participant 3	
		*‘I'm taking that as vulnerable, small, not ready to be here yet… I'm also thinking in terms of struggling about fighting infections, struggling to breathe, things‐ not necessarily things that they've contracted, things they've been that she was born with.’*—Participant 3	
		*‘I think it covers lots of things. So it's, you know, do they have breathing issues, are they, you know, how small are they? Have they got weight issues?’*—Participant 4	
		*‘When you say unwell to me is a case of he's not‐ he's not being able to be treated like a normal newborn…in whatever way, whether it's to do you know with the condition that he's got or whether he's unwell because of something that they've done, I would just consider it as him in my head not just being able to be left as a newborn baby.’*—Participant 5	
8.	Worried about changes to their condition.	*‘I assume you mean‐ my immediate thought is condition. Do we mean medical condition, like what about changes to their environmental condition? Do you mean wellness you know?’*—Participant 2	I have worried about changes to my baby's condition.
		*‘I am immediately thinking about bad changes, things getting worse.’*—Participant 3	
		*‘I'm not thinking about a specific illness no I'm thinking about kind of overall condition, like how their status is’*—Participant 3	
		*‘So when I think condition, I do think just kind of what was her overall health like’*—Participant 4	
		*‘For me, straight away makes me think of infection, potential deterioration in whatever is going on. And also changes in terms of like development changes of you know and weight like it‐ so whether‐ whether he's meeting his‐ staying on the you know, on his line on the centiles.’*—Participant 5	
9.	Concerned that interacting with them will interfere with their medical care.	*‘Again, I think that's really clear.’*—Participant 2	I have felt concerned that interacting with my baby would interfere with their medical care.
		*‘… if they start dropping their temperature or they or you dislodge a wire or a tube kind of like‐ kind of like, yeah, if you just‐ just doing what should what should feel natural to you, if that can cause problems or set them back a bit. So I'm thinking about kind of accidentally causing harm almost with that one.’*—Participant 3	
		*‘I'm thinking about literally picking her up, holding her, changing her nappy and kind of general care really.’*—Participant 3	
		*‘So I think I'm immediately thinking about equipment. I guess cus, I was thinking straight away dislodge a tube, dislodge a monitor.’*—Participant 3	
		*‘So yeah interacting in this sense in the NICU sense, I felt like holding them, picking them up because that is a thing that you can't always do, like you can always see them and like, look at them and smile, but you can't always pick them up and cuddle them. Whereas at home, obviously you can do that.’*—Participant 4	
		*‘And then medical care. Yeah, it's like the serious stuff. So it's not just feeding them, it's do they need oxygen. Do they have jaundice? Do they need, whatever kind of you know, do they need to be in the incubator? That's kind of what that makes me think of.’*—Participant 4	
		*‘…my first thought is the sort of how much have I been able to hold him and nurse him in comparison to what I would have done if he didn't have these medical things attached to him?… My brain would automatically go towards the like the physical attachments to him for me and or like being in the incubator with the lid on, you know when they're in.’*—Participant 5	
		*‘With the things that are there to get him better, it's to me it's a bit of a case of is‐ is me interacting with them more important than them. Well, to me it's not.’*—Participant 5	
10.	Frightened about developing a relationship with them because of my concern about their survival.	*‘When I think of developing a relationship, I immediately think of the word bonding. That's like my immediate response.’*—Participant 2	I have felt frightened about developing a relationship with my baby because of my concern about their survival
		*‘You see that word a lot in the hospital you know bonding with your baby… developing a relationship feels quite formal is the only thing where my instinct would be.. but I don't know if that's getting at the exact same thing as developing a relationship’—*Participant 2	
		*‘That's where my mind went to immediately when I read that I was like oh they mean bonding you know?’*—Participant 2	
		*‘[I'm thinking about] bonding… that protective instinct that kicks in when they're alone. Yeah, kind of like thinking about them as kind of like your person rather than something to look after as like, no, she is my daughter.’*—Participant 3	
		*‘So yeah, it's about bonding is what I think with that sort of bonding with the baby and getting to know them and like they know you and they cuddle into you and stuff, but I think you don't know them.’*—Participant 4	
		*‘So developing a relationship in terms of knowing his little cues, signals, the touch side of things, the physically holding him to learn, you know, his mannerisms. That just kind of getting to know the baby themselves for me.’*—Participant 5	
11.	Frightened in the hospital environment.	*‘ think that's really clear because you don't just mean it's kind of encompassing everything that is at the hospital because you're thinking about all the senses, smell, taste, you know’*—Participant 2	I have felt frightened in the hospital environment.
		*‘You know, when people just say like, I don't like hospitals, you know? And you're like, oh, why? And I'm just like, oh, I hate hospitals. I don't like the smell. I don't like the, you know, I don't like the food. I don't like the, you know, a lot of people actually do talk about the smell. And like, it's so clinical. And it's that. And it's like how I would describe that. And‐ but people just describe it as I don't like hospitals. They don't say I don't like the hospital environment.’*—Participant 2	
		*‘I'm thinking about the physical environment of the NICU, but also the wider hospital because neonatal is its own precious little bubble. But it's still it's lights, it's hand sanitiser stations, alarms.’*—Participant 3	
		*‘Yes, I'm thinking about the physical‐ so that within the neonatal unit itself, but then the wider hospital.’*—Participant 3	
		*“…it's really weird like I'd never been in there before. You go in, everything's beeping…’*—Participant 4	
		*‘So it does make me think of quite a few things like I think of I'm walking into the hospital and it's kind of, you know, going for your scans and, you know, it's all in the same place…you don't go in there for many good things.’*—Participant 4	
		*‘First thing I think of is the panic feeling of it just not being home, the experience itself of just helplessness, waiting in that, the physical environment that you just don't want to be in.’*—Participant 5	
12.	Worried that my baby is in pain.	*‘It probably should be was in pain.’*—Participant 2	I have worried that my baby was in pain.
		*‘If you're in‐ in it, it would be it's in pain but if it's we're thinking back about it would be was in pain.’*—Participant 2	
		*‘I think that's a really clearly worded question.’*—Participant 2	
		*‘This is more like a few weeks later when you're trying to feed them, and then they're crying and you don't know why they're crying now, are they in pain, have they got wind and these kind of things.’*—Participant 4	
		*‘Initially thinking of him being upset. But beyond, you know, a newborn baby's cry more a case of him suffering.’*—Participant 5	
13.	Concerned that I was unable to help them.	*‘I guess I would see that as you know a bigger question in terms of like help them generally you know.*	I have felt concerned that I was unable to help my baby.
		*But yeah, I don't know who like. Help them get better.’*—Participant 2	
		*‘So I would see that as like a as like a general thing. But I can understand some people would be like, yeah, do you mean help them get better or do you mean help them just day‐to‐day, you know, making sure they don't have a dirty nappy and they are fed.’*—Participant 2	
		*‘[I'm thinking of] mostly medical care, but also be able to offer a comfort.’*—Participant 3	
		*‘I think for me it's in the sense of like, you know, if they're hungry, give them food. If they're sad, if they need a cuddle, those are the kinds of things. But I think, you know, other parents who've had more severe outcomes might have different thoughts about that. But yeah, for me, it was just the basic, fulfilling their basic needs, basically.’*—Participant 4	
		*‘Straight away, unable to help them makes me think when the doctors and nurses are totally in control of the baby and I'm not able as his mum to step in or to have not any say, but I'm‐ I'm kind of in the back of the frame if that makes sense.’*—Participant 5	
		*‘…it's not just help as in when the doctors are there and they're completing a procedure, it's also like help when there's just me and his dad in the room and we can't really pick him up…”*—Participant 5	
14.	Worried I am not bonding with my baby.	*‘I probably read that as like in my head worried about bonding with my baby. Yeah, I think, yeah, I find bonding [a] really helpful term, I don't know. I really like that term. Yeah. Worried about bonding with my baby.’*—Participant 2	I have worried that I have not bonded with my baby in the way that I want to.
		*‘…there's a lot wrapped up in that bonding as well with feeding and yeah. Because I immediately think about when I think about bonding, I think about feeding as well.’*—Participant 2	
		*‘I'm thinking about all the things that we didn't get to do that would normally facilitate bonding, that we didn't get to.’*—Participant 3	
		*‘…kind of that instinct that kicks in that people say kicks in straight away and soon as you see your baby, it clicks.’*—Participant 3	
		*‘[When asked about the difference between 10 and 14] So ten, I think I'm thinking obviously there's a little bit bonding, but also just getting to know them as a person, fourteen I'm thinking about that really specific mother child bond that in all the baby classes and NCT classes they tell you about and it's meant to be like instinctive.’*—Participant 3	
		*‘… we could hold her as well, so we could have that and she would, like, make little noises and nuzzle, like, snuggle into me so it wasn't as big of a concern.’*—Participant 4	
		*‘First thought straight away is that initial bond that time spent together whether and‐ and to the physical bond really for me is the is what when it says I'm not bonding with my baby. I would think physically of that, you know, touch rather than‐ it doesn't make it‐ just makes me think of‐ of the lack of being able to hold.’*—Participant 5	
		*‘ [Stated that this was similar to 10 and asked to elaborate] I would be inclined to say it was the same question for me, just worded differently.’*—Participant 5	
15.	Unable to protect my baby from harm.	*‘When I see harm, I immediately think of yeah, from staff, which is bad because it's just like [from the] news… my reading of harm is‐ is thinking yeah, largely from staff.’*—Participant 2	I have felt unable to protect my baby from potential harm by medical professionals.
		*‘I'm thinking about some‐ something external going in to deliberately hurt my baby, whether that be take something away or do something to her.’*—Participant 3	
		*‘Yeah. So the immediate thought that springs to mind is about medical negligence, for some reason…’*—Participant 4	
		*‘I think as well I think that's probably the Lucy Letby case, isn't it? Because you know I have been in that environment so I can fully imagine what happened. So that is the first thing.’*—Participant 4	
		*‘I don't know whether you're alluding to harm in terms of, like safety in the hospital or whatever, but I am thinking straight away that it's a similar to unable to help in terms of it's a, it's above my‐ my skill set that it's the doctor's kind of thing.’*—Participant 5	
		*‘I'm in someone else's hands and they could cause further problems than what we're already working against, if that makes sense.’*—Participant 5	
16.	Worried about my infant's appearance.	*‘I think I‐ I would be confused about whether you meant like because you've used baby throughout and then it's infant whether you mean in the future like or whether you know like as in I'm worried about what they'll look like when they're older, you know, or whether you'll if I'm worried about what they'll look like now’*—Participant 2	I have felt worried about my baby's appearance.
		*‘If I wasn't thinking about it too much, I would immediately think, oh, because of all the wires and all of the, you know, medical equipment.’*—Participant 2	
		*‘I'm thinking about how the reasons that she ended up in NICU affected how she didn't look like a normal newborn baby.’*—Participant 3	
		*‘I'm thinking about kind of so she was very small a bit yellow covered in wires and I was‐ I'm thinking about how I was just worried that it was all just‐ I was interpreting it as a sign of that she was ill and needing help. And then it was just kind of like an indicator of things aren't going well for this little one.’*—Participant 3	
		*‘I think, I would think about this in terms of how healthy they looked, so how skinny they were, were they like pale…’*—Participant 4	
		*‘I can imagine people might think you know when they're in the incubator and you know, definitely at first because you see them and they're so little with like the tubes and things like stuck on their hands.’*—Participant 4	
		*‘So I'm thinking his appearance in terms of like whether his condition or anything has affected how, that sounds a terrible way to put it, but how normal he would look, whether it's going to have any kind of implications on his future.’*—Participant 5	
		*‘I'm kind of thinking of like whether there was any kind of deformity or that as a‐ as a like result of whatever he's‐ he's gone through that‐ that's how I see it.’*—Participant 5	
Response options		*‘There would be one or two that I would feel like I was just having to fit or where a more appropriate response would have been not applicable or I hadn't thought about, you know, yeah, not applicable. But I would obviously if I was given it, I would still answer it.’*—Participant 2	
		*‘I was surprised when I first saw there was no neutral response. But now that I'm seeing the questions that like they don't make… a neutral response would not be appropriate.’*—Participant 2	
		*‘I mean, yeah, it sometimes might be helpful, but then because you're saying in the last 7 days, I think obviously I'm thinking back quite a while now so I think they will be fine…I'd say keep it the same unless there's a real rationale to change it.’*—Participant 4	
		*‘So to me, not very often would be like once in 7 days, often would be like maybe four or five times in 7 days, like in per day kind of thing.’*—Participant 5	
Comments on the scale		*‘And also the questions are quite‐ they really make you think about like which is I think such a like a huge support for it that they felt really on the nose, you know? And it's like, gosh, yeah, that's exactly how I felt at the time.’*—Participant 2	
		*‘So then it makes you think about yes, obviously you're thinking about the time when you're in NICU, but you were‐ you're thinking now about how you thought then about the future, you know.’*—Participant 2	
		*‘…because it's hitting‐ it's like long enough to kind of be like, it's not skimming it over experience because it's such a complex experience.’*—Participant 3	
		*‘…we've got friends that were full term NICU babies, preemie NICU babies. Yeah, I can see how it applies to them as well.’*—Participant 3	
		*‘[Asked if relevant post‐discharge] So if you're thinking about you're, you're asking somebody to reflect on their experience. Especially if, let's say you've got‐ you're out of NICU, you've gone home, but you've needed input from the perinatal mental health team. It's yeah, because rather than just kind of both postpartum, it's a specific experience.’*—Participant 3	
		*‘I would say it's probably it's manage‐ it's manageable without it being too intense, so it's not too long winded.’*—Participant 5	
		*‘My‐ my main worry when we were on the ward for us as a family was that when we got home is my anxiety for his health and his survival gonna ruin the whole experience of having a newborn, and I know you've got the readmission to hospital and the fear of survival, but there could maybe be a question of more geared to when you get home of that‐ that worry of what do we do when we go home, like as a as a mum or as parents is like, do you get not necessarily about the condition itself or about them surviving, but more about the fear of that hospital environment has gone. And it's just us.’*—Participant 5	

Concerning the PSAS‐PTB, the only change to the scale was to the item *‘I have worried that I will do further harm to my infant if I interact with them because of their prematurity’* was changed to *‘I have worried that I will harm my baby if I interact with them because of their prematurity’* because participants were inferring that the question implied they had purposely harmed their infant.

With regard to the PSAS‐NICU, whilst most participants did consider the *‘When my baby was in NICU, I am/have felt…’* preamble when responding, most were only considering when they were in the NICU. As such, it was agreed to remove this preamble and change items to be in the past tense, considering the 7‐day recall period that was already orienting participants. During the interviews, participants agreed the items could be answered whilst in NICU and upon discharge. In terms of individual item changes: *‘Concerned about them needing support from medical equipment’*, was changed to *‘I have felt concerned about my baby needing continuous support from medical equipment’* to ensure the item was applicable to both the NICU and discharge home. Furthermore, *‘Worried I am not bonding with my baby’* was amended to *‘I have worried that I have not bonded with my baby in the way that I want to.’* Although one participant expressed this was similar to another question, they were the only participant to express this interpretation and others distinguished between them, so this was retained. Finally, in the question about appearance, the word *‘infant’* was changed to *‘baby’* to be consistent with the remainder of the questionnaire, and because participants were mistakenly thinking of longer time frames for this question.

### 3.4 Protocol for Study Stage 4: Psychometric Validation of the PSAS‐PTB and PSAS‐NICU via Large Scale On‐Line Survey

The final step in the development of any psychometric scale is to ensure comprehensive validity and reliability testing. This will be implemented with both proposed scales—for which the final items at this stage, can be found in Tables 6 and 7—before widespread implementation in future research. It is important to note that these items must be subjected to psychometric evaluation and so may differ from the final resultant items of each scale.

**TABLE 6:** Iterations of the PSAS‐PTB after each stage of the study.

Postpartum specific anxiety scale—Preterm birth [PSAS‐PTB]		
PPIE groups	Expert panel	Cognitive interviews
I have worried I cannot provide the right care for my premature baby	I have worried I cannot provide the right care for my premature baby	I have worried I cannot provide the right care for my premature baby
I have felt that my baby being born prematurely was my fault	I have felt that my baby being born prematurely was my fault	I have felt that my baby being born prematurely was my fault
I have worried about my baby becoming ill because of their prematurity	I have worried about my baby becoming unwell because of their prematurity	I have worried about my baby becoming unwell because of their prematurity
I have worried about my baby's future development because of their prematurity	I have worried about my baby's future development because of their prematurity	I have worried about my baby's future development because of their prematurity
I have worried about my baby's future quality of life because of their prematurity	I have worried about my baby's future quality of life because of their prematurity	I have worried about my baby's future quality of life because of their prematurity
I have not been able to stop thinking about the size of my baby	*Item removed*	
I have worried about my future quality of life because of my baby's prematurity	I have worried about my future quality of life because of my baby's prematurity	I have worried about my future quality of life because of my prematurity
I have worried that my baby is not gaining enough weight	I have worried that my baby is not gaining enough weight because of their prematurity	I have worried that my baby is not gaining enough weight because of their prematurity
I have worried about the size of my baby	I have worried about the small size of my baby	I have worried about the small size of my baby
I have worried that I will do further harm to my infant if I interact with them because of their prematurity	I have worried that I will do further harm to my infant if I interact with them because of their prematurity	I have worried that I will harm my baby if I interact with them because of their prematurity
I have worried about my infant's appearance	*Item removed*	
I have felt uncertain about my baby's future	*Item removed*	
	I have worried about the low birthweight of my baby	I have worried about the low birthweight of my baby

*Note: N/B.* The items in each column represent the final versions after each stage which were then progressed to the next study stage.

**TABLE 7:** Iterations of the PSAS‐NICU after each stage of the study.

Postpartum specific anxiety scale—Neonatal intensive care unit [PSAS‐NICU]		
PPIE groups	Expert panel *(N.B* this version included the preamble: ‘*When my baby was in NICU, I am/have felt…’)*	Cognitive interviews
I have worried about providing additional medical care for my baby	Worried about having to provide additional medical care for them	I have worried about providing additional medical care for my baby
I have worried about my baby being re‐admitted to hospital	Concerned about them being re‐admitted to hospital in the future	I have felt concerned about my baby being readmitted to hospital in the future
I have worried about the survival of my infant	Frightened about their survival	I have felt frightened about my baby's survival
I have worried about medical equipment on or near my baby	Concerned about them needing support from medical equipment	I have felt concerned about my baby needing continuous support from medical equipment
I have worried about how much time I have been able to spend with my baby	Worried about how much time I have been able to spend with them	I have worried about how much time I have been able to spend with my baby
I have worried about my baby becoming exposed to infection in hospital settings	Frightened about my baby becoming exposed to infection	I have felt frightened about my baby becoming exposed to infection
I have not been able to stop thinking about how ill my baby is	Not been able to stop thinking about how unwell they are	I have not been able to stop thinking about how unwell my baby is
I have repeatedly worried about changes to my baby's condition	Worried about changes to their condition	I have worried about changes to my baby's condition
I have repeatedly asked about the status of my baby's health	*Item removed*	
I have worried that interacting with my baby will interfere with their medical care	Concerned that interacting with them will interfere with their medical care	I have felt concerned that interacting with my baby would interfere with their medical care
I am worried about developing a relationship with my baby because I am concerned about their survival	Frightened about developing a relationship with them because of my concern about their survival	I have felt frightened about developing a relationship with my baby because of my concern about their survival
I have felt frightened in the NICU environment	Frightened in the hospital environment	I have felt frightened in the hospital environment
I have worried that my baby is in pain	Worried that my baby is in pain	I have worried that my baby was in pain
I have worried that I do not get alone time with my baby	*Item removed*	
I have felt unable to protect my baby from harm	Unable to protect my baby from harm	I have felt unable to protect my baby from potential harm by medical professionals.
	Concerned that I was unable to help them	I have felt concerned that I was unable to help my baby
	Worried I am not bonding with my baby	I have worried that I have not bonded with my baby in the way that I want to
	Worried about my infant's appearance	I have felt worried about my baby's appearance

*Note: N/B.* The items in each column represent the final versions after each stage which were then progressed to the next study stage.

Eligible participants will be required to be over the age of 18 with a good understanding of the English language, who either have a live infant born prematurely (< 37 weeks' gestation) now aged between 0 and 12 months corrected age who has/has not previously spent time in the NICU, or a live infant born at term now aged between 0 and 12 months who has previously spent time in the NICU, but was not born prematurely. Women with a severe mental health disorder or learning disability will not be eligible to participate in the study due to an abundance of caution surrounding capacity to consent and to signpost and/or support if required, in an anonymous, on‐line format, which the study will adopt. Participants fulfilling eligibility criteria will be recruited via social media (e.g., X [formerly Twitter], Facebook, Reddit, Instagram, etc.). Participants will complete the PSAS‐RSF (16 items) in addition to the PSAS‐PTB (10 items) and/or the PSAS‐NICU (16 items). We aim to recruit approximately 200 participants for both the EFA and CFA as a minimum (Bryant and Yarnold 1995; Hair et al. 1998). This is because we expect there to be high factor loadings and high communalities between items. These sample sizes will also be adequate for the assessment of internal reliability Participants will access the study on‐line, hosted via Qualtrics. After reading the information sheet and filling in a consent form participants will firstly be asked some initial screening questions to ensure they meet the eligibility criteria. Participants will then complete demographic questions (e.g., age, ethnicity, occupation, gestational age, and length of stay in NICU, if applicable). They will then complete the PSAS‐PTB (if applicable), the PSAS‐NICU (if applicable), and the PSAS‐RSF (Davies et al. 2021). A battery of other measures will then be completed, including items from the Edinburgh Postnatal Depression Scale (EPDS; Cox et al. 1987), the Postpartum Bonding Scale (PBQ; Brockington et al. 2001), the Revised Infant Temperament Questionnaire (RITQ; Carey and McDevitt 1978), the Perceived Maternal Parenting Self‐Efficacy Scale (PMPSES; Barnes and Adamson‐Macedo 2007). For those who have indicated their infant has spent a period of time in the neonatal intensive care unit, the Parental Stressor Scale: Neonatal Intensive Care Unit (PSS:NICU; Miles et al. 1993). Participants will then be shown a debrief sheet with an opportunity to enter a prize draw for a £25 voucher.

Additionally, participants will be asked if they would like to take part in a follow‐up study 2 weeks later, to establish test‐retest reliability. Participants who complete the secondary survey 2 weeks later will re‐complete the PSAS‐PTB (if applicable), PSAS‐NICU (if applicable), the PSAS‐RSF, the RITQ, and the PBQ, again.

Due to responses being 4‐level ordinal, the EFA will be conducted on the polychoric correlation matrix, and the CFA will use a diagonally weighted least squares estimator, or maximum likelihood with robust standard errors, as appropriate. Internal reliability will be assessed using McDonald's Omega total (for subscales) and Omega hierarchical to assess reliable variance in the total scale score. Test re‐test reliability will be calculated using intraclass correlations. Convergent and divergent validity of the scales will be tested using correlations.

## 4 Discussion and Future Directions

The development of the PSAS‐PTB and PSAS‐NICU has consisted of a comprehensive, multi‐stage process with many strengths. To our knowledge, this is the first postpartum‐specific measure of anxiety which has been adapted specifically for use in mothers of preterm infants and those who have had infants admitted to the NICU. The development had input from a range of stakeholders, include clinicians, psychologists, and experts by lived experience—across a multi‐stage development—which has not previously been documented for a perinatal mental health psychometric scale. Efforts will be made to recruit participants from diverse backgrounds during the validation study for which the above protocol outlines, as we are cognisant the development has received input from mothers from higher socio‐economic backgrounds than the national average, particularly in the cognitive interview study. This programme of work describes the process for the development of, and the planned methodology for the validation of the PSAS‐PTB and the PSAS‐NICU. Items on the newly proposed scales have had input from a range of stakeholders, contributing to a range of comprehensive testing which has ensured their relevance and understanding in these specific populations. Future research must now validate both scales before they are used in research, whilst further validation will be required for use in clinical settings, including analyses with the gold standard clinical interview. The ability of the PSAS‐NICU to be used as a ‘Velcro’ subscale, either alone or in conjunction with other derivatives of the PSAS, will further enhance its application. Further comprehensive translation will be required for use in different countries and cultural settings.

## Author Contributions

**Semra Worrall:** conceptualisation, investigation, data curation, formal analysis, visualisation, writing – original draft, methodology, project administration, software. **Paul Christiansen:** conceptualisation, writing – review and editing, supervision, resources, validation. **Asma Khalil:** conceptualisation, supervision, writing – review and editing, resources, validation. **Victoria Fallon:** conceptualisation, supervision, writing – review and editing, resources, validation. **Sergio A. Silverio:** conceptualisation, supervision, writing – review and editing, validation, resources, writing – original draft, methodology, formal analysis.

## Ethics Statement

The study received full ethical approval from the University of Liverpool Institute of Population Health Research Ethics Committee (ref:‐ 12880).

## Conflicts of Interest

The authors declare no conflicts of interest.

## Supporting information


Supporting Information S1

## Data Availability

The data that support the findings of this study are available on request from the corresponding author. The data are not publicly available due to privacy or ethical restrictions.
