# Supplementary material for: A Protocol for the Development and Validation of the Postpartum Specific Anxiety Scale—Preterm Birth [PSAS‐PTB] and the Postpartum Specific Anxiety Scale—Neonatal Intensive Care Unit [PSAS‐NICU]
Source: Int J Methods Psychiatr Res. 2025 Aug 9;34(3):e70032. doi: 10.1002/mpr.70032 (PMC12335222; doi:10.1002/mpr.70032)
Supplement: Supplementary file 1 — Supporting Information S1 [file MPR-34-e70032-s001.docx]

| **Supplementary Table 1. PSAS-PTB items to retain, remove, or modify with reasons (N=16).** | | | | | | | |
| --- | --- | --- | --- | --- | --- | --- | --- |
| **Item** | **Retain (N/%)** | **Reasons for Retaining** | **Remove (N/%)** | **Reasons for Removal** | **Modify (N/%)** | **Reasons for Modification** | **Additional Items** |
| I have worried I cannot provide the right care for my premature baby | 14 (87.5%) | *Confidence*  *Fine item for mothers of premature infants*  *Focus on aftercare is good, prem-health specific*  *Immediate source of concern*  *Including this item can be very pertinent because mothers of premature babies often face unique challenges, including providing adequate care given the baby’s additional health needs.*  *May not be a general response*  *Mothers of preemis may have doubts about their capacity to take care*  *Raises concerns about premature infant care*  *Relevant anxiety*  *Relevant for a mother who might perceived herself as good enough as the “let” the infant to be born prematurely*  *Vulnerability of baby* | 0 (0.0%) |  | 1 (6.3%) | *Checked in error – would not modify.* | *No*  *No*  *No* |
| I have felt that my baby being born prematurely was my fault | 10 (62.5%) | *Feelings of guilty are common*  *Fine item for mothers of premature infants*  *Including this item can be very pertinent because it addresses common feelings of guilt and self-blame that many mothers of premature babies experience*  *not usually reasonable*  *relevant as the event might be perceived as mother’s responsibility*  *SO common and can prevent bonding effect relationships*  *Talk about the mother’s guilt*  *Taps into guilt and shame* | 0 (0.0%) |  | 1 (6.3%) | *I’m anxious something I did led to my baby’s early birth.* |  |
| I have worried about my baby becoming ill because of their prematurity | 11 (68.8%) | *Fine item for premature mothers*  *Health-specific concern*  *Immediate source of anxiety for a vulnerable infant.*  *Including this item can be very pertinent because premature babies are at a higher risk for various health issues, including respiratory problems, infections, and developmental delays, due to their early birth.*  *Preemies may have deficits, illnesses etc*  *Relevant anxiety about baby’s health*  *She points out concerns about her baby’s future* | 1 (6.3%) | *The word ‘ill’ is unclear* | 0 (0.0%) |  |  |
| I have worried about my baby’s future development because of their prematurity | 13 (81.3%) | *As stated above*  *Could unknown future, excessively worry*  *Fine item for premature mothers*  *I like how you’ve broken down developmental, QoL, and appearance*  *Including this item can be very pertinent because prematurity can lead to a range of potential developmental delays and challenges, from cognitive and motor skills to social and emotional development*  *May depend on degree of prematurity*  *Relevant anxiety*  *Relevant anxiety about baby’s health*  *She points out concerns about her baby’s future* | 1 (6.3%) | *Normal response* | 0 (0.0%) |  |  |
| I have worried about my baby’s future quality of life because of their prematurity | 11 (68.8%) | *As stated above*  *Fine item for premature mothers*  *Including this item can be very pertinent because prematurity can lead to a range of potential developmental delays and challenges, from cognitive and motor skills to social and emotional development.*  *Relevant anxiety*  *She points out concerns about her baby’s future*  *Unknown future, excessively worry*  *Well operationalised.* | 1 (6.3%) | *Normal response* | 0 (0.0%) |  |  |
| I have not been able to stop thinking about the size of my baby | 8 (50.0%) | *Fine item for premature mothers*  *Including this item can be very pertinent because for premature babies, size can be a significant indicator of health and development. Babies born prematurely often have lower birth weights and may grow at a slower rate initially. Mothers might worry about how their size and growth could affect their baby’s immediate and long-term health.*  *Relevant anxiety*  *Relevant anxiety about the baby’s health*  *Signals concern about the baby’s health* | 4 (25.0%) | *Repetition of item 9*  *Seems more linked to trauma than anxiety*  *The word ‘size’ is unclear* | 0 (0.0%) |  |  |
| I have worried about my future quality of life because of my baby’s prematurity | 7 (43.8%) | *Fine item for premature mothers*  *Good that you cover maternal concerns about satisfaction as well*  *Including this item can be very pertinent because the potential for ongoing health issues, developmental challenges, or special needs can significantly impact a mother’s outlook on her future life, including her personal, social, and professional aspects.*  *Relevant anxiety*  *She points out concerns about her baby’s future*  *Unknown future, excessively worry* | 1 (6.3%) | *I think parents are more concerned about their baby’s wellbeing rather than th* | 1 (6.3%) | *I think it should be more specific of the aspects mum is worried about* |  |
| I have worried that my baby is not gaining enough weight | 11 (68.8%) | *Due to their prematurity (needs rephrasing to make prem-specific and to marry up with other included scale items).*  *Fine item for premature mothers*  *Including this item can be very pertinent because weight gain is a critical indicator of health and development in newborns, especially in premature babies who often start life with low birth weights.*  *Major issue*  *Reflects concern for small/preterm infant.*  *Relevant anxiety*  *Relevant anxiety about baby’s health*  *Signals concern about the baby’s health* | 0 (0.0%) |  | 2 (12.5%) | *…due to their prematurity.*  *Could worry regardless if preterm* |  |
| I have worried about the size of my baby | 7 (43.8%) | *Fine item for premature mothers*  *Major issue*  *Relevant anxiety about the baby’s health*  *Same as 8, needs to be more prem-specific*  *Signals concern about the baby’s health* | 2 (12.5%) | *Concerns about a baby’s size can be very common among all new parents, not uniquely indicative of mothers of premature babies or those experiencing pathological anxiety.* | 3 (18.8%) | *…due to their prematurity*  *Add context. Because my baby is small I don’t hold or wash them etc.*  *Could worry about this regardless if preterm* |  |
| I have worried that I will do further harm to my infant if I interact with them because of their prematurity | 10 (62.5%) | *Anxiety could result from the ideal that parent unable to look after child adequately*  *Could influence behaviour*  *Fear of harm/not being good enough will likely be a big driver here.*  *Fine item for premature mothers*  *Including this item can be very pertinent because this concern directly taps into the anxiety surrounding the care of a premature infant, who is often perceived as more fragile compared to full-term babies. Mothers might fear that normal handling or interaction could lead to complications or setbacks in their infant’s health, reflecting deep-seated anxieties about causing unintentional harm.*  *Raises concern about premature infant care*  *Relevant anxiety* | 1 (6.3%) | *I think the item is not clearly described.* | 0 (0.0%) |  |  |
| I have worried about my infant’s appearance | 4 (25.0%) | *Good.* | 5 (31.3%) | *I am not sure if it is related to prematurity*  *Not very relevant*  *This seems an unusual worry*  *Worry about an infant’s appearance can be a common concern for many parents. This worry might not necessarily indicate an anxiety disorder but rather a normal sensitivity to societal expectations or personal hopes for the baby.* | 3 (18.8%) | *…due to their prematurity.*  *Depends on issues but could be seen as an odd question*  *I think it should be more specific on the aspects mum is worried about* |  |
| I have felt uncertain about my baby’s future | 10 (62.5%) | *As explained above*  *Fine item for premature mothers*  *Including this item can be very pertinent because uncertainty about the future is a significant concern for mothers of premature babies, who often face uncertain health outcomes.*  *Relevant anxiety*  *She points out concerns about her baby’s future*  *Underlying concern about whether baby could end up disabled or die.*  *Unknown future, excessively worry* | 2 (12.5%) | *Already captured in other items.*  *Normal response* | 0 (0.0%) |  |  |

| **Supplementary Table 2. PSAS-NICU items to retain, remove, or modify with reasons (N=16).** | | | | | | | |
| --- | --- | --- | --- | --- | --- | --- | --- |
| **Item** | **Retain (N/%)** | **Reason for Retaining** | **Remove (N/%)** | **Reason for Removal** | **Modify (N/%)** | **Reason for Modification** | **Additional Items** |
| I have worried about providing additional medical care for my baby | 10 (62.5%) | *All seem important and very relevant*  *Basic concern about providing care*  *High relevance*  *It is a major concern for mothers of premature babies*  *Talk about worrying about not being able to care for or protect the baby.* | 0 (0.0%) |  | 2 (12.5%) | *…since they have been admitted to NICU.*  *This is unclear – who is providing additional medical card? What is additional medical care?* | *I would add some more around being able to be re engaged or caring for the infant in the NICU*  *Need to cover bonding as lack of bonding between mother/parents and baby could be a major source of concern.*  *No*  *No, I find the questionnaire very complete.* |
| I have worried about my baby being re-admitted to hospital | 11 (68.8%) | *All seem important and very relevant*  *Anxiety about baby’s health*  *High relevance*  *It is a major concern for mothers of premature babies*  *Talk about worrying about not being able to care for or protect the baby.* | 1 (6.3%) | *Normal worry* | 0 (0.0%) |  |  |
| I have worried about the survival of my infant | 13 (81.3%) | *All seem important and very relevant*  *Concern about the baby’s health*  *High relevance*  *It is a major concern for mothers of premature babies*  *Primary concern of all parents whose babies are born early*  *Relates to mothers anxiety* | 1 (6.3%) | *Normal worry* | 1 (6.3%) | *…since they have been admitted to NICU.* |  |
| I have worried about medical equipment on or near my baby | 7 (43.8%) | *All seem important and very relevant*  *Discusses concerns about the healthcare environment*  *High relevance*  *It is a major concern for mothers of premature babies* | 1 (6.3%) |  | 2 (12.5%) | *…since they have been admitted to NICU.*  *I would re-work: I am worried about my baby being hooked up to medical equipment – focus on the baby, not the equipment* |  |
| I have worried about how much time I have been able to spend with my baby | 8 (50.0%) | *All seem important and very relevant*  *High relevance*  *It is a major concern for mothers of premature babies*  *Talk about worrying about not being able to care for or protect the baby.* | 0 (0.0%) |  | 1 (6.3%) | *…since they have been admitted to NICU.* |  |
| I have worried about my baby becoming exposed to infection in hospital settings | 8 (50.0%) | *All seem important and relevant*  *Discusses concerns about the healthcare environment*  *High relevance*  *It is a major concern for mothers of premature babies* | 1 (6.3%) | *Normal worry* | 1 (6.3%) | *…since they have been admitted to NICU.* |  |
| I have not been able to stop thinking about how ill my baby is | 13 (81.3%) | *All seem important and very relevant*  *Anxiety about baby’s health*  *Concern about the baby’s health*  *High relevance*  *It is a major concern for mothers of premature babies*  *Primary concern relating to baby’s condition*  *Relates to mothers anxiety* |  |  |  |  |  |
| I have repeatedly worried about changes to my baby’s condition | 11 (68.8%) | *All seem important and very relevant*  *Concern about the baby’s health*  *High relevance*  *It is a major concern for mothers of premature babies*  *Relates to mothers anxiety*  *Relating to baby’s survival.* | 2 (12.5%) | *I don’t think you need both Q8 and Q9. Would select one of them.* | 1 (6.3%) | *…since they have been admitted to NICU.* |  |
| I have repeatedly asked about the status of my baby’s health | 11 (68.8%) | *All seem important and very relevant*  *Concern about the baby’s health*  *High relevance*  *It is a major concern for mothers of premature babies*  *Relates to mothers anxiety* | 1 (6.3%) | *I don’t think you need both Q8 and Q9. Would select one of them.* | 1 (6.3%) | *…since they have been admitted to NICU.* |  |
| I have worried that interacting with my baby will interfere with their medical care | 11 (68.8%) | *All seem important and very relevant*  *Anxiety due to lack of information and sense of being inadequate*  *Could influence behaviour*  *Discusses concerns about the healthcare environment*  *High relevance*  *It is a major concern for mothers of premature babies*  *Relates to mothers anxiety* | 1 (6.3%) |  | 1 (6.3%) | *…since they have been admitted to NICU.* |  |
| I have worried about developing a relationship with my baby because I am concerned about their survival | 13 (81.3%) | *All seem important and very relevant*  *High relevance*  *It is a major concern for mothers of premature babies*  *Relates to mothers anxiety*  *Relating to baby’s survival and lack of bonding*  *Talk about worrying about not being able to care for or protect the baby* | 0 (0.0%) |  | 1 (6.3%) | *…since they have been admitted to NICU.* |  |
| I have felt frightened in the NICU environment | 10 (62.5%) | *All seem important and very relevant*  *Discusses concerns about the healthcare environment*  *High relevance*  *It is a major concern for mothers of premature babies*  *NICU is really frightening, particularly the ICU.* | 0 (0.0%) |  |  |  |  |
| I have worried that my baby is in pain | 10 (62.5%) | *All seem important and very relevant*  *Anxiety due to lack of information and about baby’s health*  *High relevance*  *It is a major concern for mothers of premature babies*  *Talk about worrying about not being able to care for or protect the baby.* | 0 (0.0%) |  | 2 (12.5%) | *…since they have been admitted to NICU.*  *I would add: I have worried that my baby is in pain and I cannot help.* |  |
| I have worried that I do not get any alone time with my baby | 7 (43.8%) | *All seem important and very relevant*  *High relevance*  *It is a major concern for mothers of premature babies*  *Talk about worrying about not being able to care for or protect the baby.* | 4 (25.0%) | *It’s too vague, it might depend on the NICU*  *This does not seem important* | 2 (12.5%) | *…since they have been admitted to NICU.*  *I worry that I am not bonding with my baby.* |  |
| I have felt unable to protect my baby from harm | 15 (93.8%) | *All seem important and very relevant*  *Anxiety about not being a “good enough mother” and cause of prematurity*  *High relevance*  *It is a major concern for mothers of premature babies*  *Relates to mothers anxiety*  *Reliant upon healthcare workers,*  *Talk about worrying about not being able to care for or protect the baby.* | 0 (0.0%) |  | 1 (6.3%) | *…since they have been admitted to NICU.* |  |

| **Supplementary Table 3. General scale comments (N=16)** | |
| --- | --- |
| **Question** | **Comments** |
| What is the ideal length for a scale of this nature? | *10-12 questions feels sufficient to gather enough clinical information but does not overload parents who’s capacity is limited*  *12 items*  *15 items – not too long*  *15-16 items is a ideal length because mother hav not many times to answer questionnaires*  *15-20. Especially once home as so tired and busy*  *20 items – can be integrated in research and routine care*  *5 mins – quick and easy to administer.*  *Around 10-items, to prevent participant burden and allow for other testing alongside other psychometric measures in research. Easier clinical and community use.*  *I would avoid more than 15 questions for parents.*  *If asking busy new parents then as short as possible while retaining as much info as you require!*  *It’s a difficult question, I suggest ideal 24 items not mor than 32.*  *Not more than 20 questions. Too long can be a deterrent to complete*  *Not take longer than 10 minutes to complete,* |
| Should the measure consider functioning as well as frequency of anxiety? | *Arguably yes – if high anxiety but not frequent then that is a different picture to low anxiety every day*  *Not a bad idea but would suggest that it is developed to marry up with other affect scales like the PSAS and EPDS for consistency. Also, if a mum feels these things frequently but lower grade, how do you gauge importance/intensity against someone who experiences these things less frequently but more intensely? Not sure it necessarily offers additional insights above intensity.*  *Only the frequency so as not to make it too long*  *Functioning may highlight specific difficulties relating to relationship with infant which may not be picked up within frequency based questions*  *Yes; functioning a key part of diagnosing a mental disorder.*  *Yes*  *Yes*  *Frequency should be enough*  *yes as it might give an idea of the degree of anxiety*  *Yes, both. You can have a lot of anxiety at one time point as parents of preterm infants, but this may vary as the infant grows beyond the preterm stage. there may also be differences in anxiety levels depending on when the infant was born preterm.*  *Yes. In case of frequency it's important to quantify how often anxious thoughts or feelings occur to gauge the intensity of the anxiety and its pervasiveness in the mother's life. In case of functioning because understand how anxiety affects a mother's ability to function daily is crucial. Anxiety that significantly impairs functioning suggests a more severe disorder that requires targeted intervention.*  *yes, I think it's important to know how long symptoms are lasting*  *Yes*  *I think it should have a separate question which asks whether the level of anxiety is altering their ability to function as a marker of severity.*  *Certainly yes, frequency is not a perfect synonym of functioning or impact.* |
| The measure only asks participants about the anxiety during the previous 7 days. Is this time period appropriate to capture anxiety in this population? | *Yes*  *Yes*  *yes, if have a tight inclusion criteria of who you are asking in a study so that it is not mixed together with parents whose babies were born preterm a while ago*  *Yes –*  *I am unsure*  *Yes*  *Yes*  *I would say "since your premature infant was born"*  *yes, it hikn so*  *yes*  *yes*  *I think it's important to catch when the questionnaire is been administered ... a baby may be in NICU for a long time so it's important to have this information ...*  *Yes*  *It depends on when it would be administered. I think other scales tend to say the prior 14 days e.g. GAD-7.*  *Maybe better to evaluate the anxiety in the last 15 days, to get a more general idea of the emotional state over time.* |
| The current scoring of the measure is on a scale from 1-4 (1=not at all, 2=not very often, 3=often, 4=almost always). Is this scoring appropriate? | *A bit subjective, can you operationalise a bit more e.g., break it up by number of days experienced the worry so a 8-point Likert scale from 0-7??*  *Yes*  *Yes – do not have a middle option (odd number of options)*  *Yes*  *Yes*  *Yes*  *I would add sometimes between not at all and often*  *could add 'sometimes- a big jump from not at all to often. Very often and almost always is also quite close so could be merged if still want to keep a scale of 1 to 4*  *yes*  *yes*  *yes*  *Yes, although the frequency in GAD-7 might be easier to conceptualise, never, 1-2 days, most days, every day?*  *I would prefer "almost never", "sometimes", "often", "very often", "almost always", It seems to me a scale with more homogeneous distances, but I understand if you want to remain consistent with the previous PSAS, then perhaps I would only change "almost never" to be consistent with "almost always".* |

| **Supplementary Table 4. PSAS-RSF relevance for use in mothers of premature infants (rated from 1=not at all relevant, 4=highly relevant), with comments on items. N=16** | | |
| --- | --- | --- |
| **Item** | **Mean(SD)** | **Comments** |
| I have worried about the length of time my baby sleeps | 2.75(0.78) | *Not NICU-specific. This is applicable to all infants.*  *If in NICU may be less relevant*  *Sleep is very important for Neonates - however, I wonder whether it is unlikely to be a top priority for mothers in the early stages of time home with a premature infant.*  *Premature infants may have sleep problems that cause anxiety*  *It seems relevant to detect anxiety in a new mother, not specifically of a mother of a baby born prematurely*  *Especially post discharge from NICU. May be developing postnatal OCD, and also are they staying awake to watch baby.* |
| I have felt unable to juggle motherhood with other responsibilities | 3.00(0.89) | *Not NICU-specific. This is applicable to all infants.*  *Premature infants may require particular (sometimes prolonged) care and surveillance*  *It seems relevant to detect anxiety in a new mother, not specifically of a mother of a baby born prematurely*  *Most new mothers can’t juggle anything*  *This could reflect other issues as well as a premature infant, such as looking after siblings of the affected infant, or even returning to work / other non-paid roles.* |
| I have worried that my baby is picking up on my anxieties | 3.06(0.77) | *As almost all mothers, mothers of prematures may be worry about transmitting their stress and anxiety to the baby*  *It seems relevant to detect anxiety in a new mother, not specifically of a mother of a baby born prematurely*  *I think this would reflect the anxiety the mother perceives that she has.* |
| I have had negative thoughts about my relationship with my baby | 3.50(0.82) | *may be more relevant for depression*  *Separation after birth may cause negative thoughts, bonding difficulties, feelings of distance with the baby (not my baby, a stranger...)*  *It seems relevant to detect anxiety in a new mother, not specifically of a mother of a baby born prematurely*  *Depending on whether the baby has been in the NNU - this may be particularly relevant*  *Bonding*  *I think this might reflect depression or possibly psychosis.* |
| I have worried about my baby’s milk intake | 3.38(0.72) | *May be justified worry-not a sign of anxiety*  *Feeding is a major issue here*  *It might be more relevant than the previous items as baby born prematurely can be perceived as not be mature enough for feeding or more a risk of stay underweight*  *As before. The loss of NICU support.*  *Similar to the first question about sleeping, this reflects concern about baby's normal activities.* |
| I have worried that my baby will stop breathing whilst sleeping | 3.81(0.40) | *Also a major issue here*  *This fear can be common to mothers, but also particularly strong for mothers who already feared their baby could have died because born too soon*  *As the baby is vulnerable, this may feel important*  *As above. And staying awake to watch baby.*  *I think this is a concern about baby's fundamental wellbeing.* |
| I have felt that I have had less control over my day than before my baby was born | 3.13(0.89) | *Imagine uncertainties around infant's health and lack of control will be big driving forces for anxiety in this sub-population.*  *may just be reality rather than a sign of anxiety*  *Feeling things are out of control can be experienced by mother whose babies are born unexpectedly sooner than expected*  *It depends when this questionnaire was going to be used. Answers while a baby is on a neonatal unit could differ from those after discharge home.* |
| I have repeatedly checked on my sleeping baby | 3.69(0.48) | *Could be more prem-specific.*  *may be done by most mothers- not necessarily a song sign of anxiety*  *Major issue also*  *Fear of cot death can be common to mothers, but also particularly strong for mothers who already feared their baby could have died because born too soon*  *Good to know frequency*  *Reflects the degree to which there is concern for baby's wellbeing / basic processes in Q6.* |
| I have felt that my baby would be better cared for by someone else | 3.13(0.89) | *Mothers of prematures may feel less competent than others*  *This fear can be experienced by many mothers, but mother born prematurely could perceive themselves as "not good enough" because they did not manage to keep their baby inside them long enough*  *Bonding. Self doubt*  *I think this might reflect postnatal depression symptoms rather than anxiety.* |
| I have worried more about my finances than before my baby was born | 2.75(0.93) | *relevant depending of the parents finances and the health system*  *This seems unrelated as common to many parents, not necessarily to parents of premature infants*  *I don't think this is a good measure of postnatal anxiety, because respondents may not be in control of their money / could be a partner.* |
| I have worried about my baby’s weight | 3.56(0.73) | *May be justified*  *Major issue for preemies*  *This worry is highly linked to a baby born sooner, smaller and more underweight than expected by mothers*  *Reflects the degree to which there is concern for baby's wellbeing* |
| I have worried that I am not going to get enough sleep | 2.88(0.81) | *relevant if the baby has special needs, has irregular sleep rythms*  *Common in all parents*  *I don't think this would reflect anxiety in postnatal women whose babies are on the neonatal unit, but may do so when they are at home.* |
| I have felt frightened when my baby is not with me | 3.56(0.63) | *Major issue for parents of preemies (separation anxiety)*  *This fear might be link to the feeling of not being in control experienced by mothers of premature infants*  *I think this would be an important issues, particularly when parents are separated from baby's whilst they are in the neonatal unit.* |
| I have worried about getting my baby into a routine | 2.44(0.81) | *could be normal, not a sign of anxiety*  *Might be common in all parents*  *I think this might be relevant particularly when parents are on the neonatal unit, because so much of the activity is undertaken by others and the timeframe for events is not dictated by parents.* |
| I have worried about my baby being accidentally harmed by someone or something else | 3.31(0.70) | *It might be common to many parents, but mothers of premature infants may particularly fear that something bad and out of their control might happen to their infants*  *Maternal OCD*  *Before the recent trial of Lucy Letby and the subsequent Thirwall inquiry I would have said that this was not relevant at all to parents whose babies were born preterm and were in hospital care, but I know think that this is an issue for parents, though it may be transient.* |
| I have worried I will not know what to do when my baby cries | 3.06(0.93) | *Common for many parents*  *I think this is a common concern for parents, but I think could be a good measure of how much anxiety a parent has about their role.* |

| **Supplementary Table 5. PSAS-RSF items to retain, remove, or modify with reasons. (N=16)** | | | | | | | |
| --- | --- | --- | --- | --- | --- | --- | --- |
| **Item** | **Retain N/%** | **Reasons for Retaining** | **Remove N/%** | **Reasons for Removal** | **Modify N/%** | **Reasons for Modification** | **Additional Items** |
| I have worried about the length of time my baby sleeps | 4 (25%) | *Fine item for any mother*  *Sleep difficulties are frequent in preemies*  *Talks about baby care concerns* | 5 (31.3%) | *All new parents become obsessed with sleep!*  *Could be a normal reaction to having a prem baby*  *Not unusual concern*  *Removing this item can be pertinent because of its redundancy with other items. As the scale already includes other items that are more directly measure anxiety-related sleep concerns, such as excessive checking behavior, this item might be redundant. Removing it could streamline the scale without losing valuable diagnostic information.* | 1 (6.3%) | *Need to make prem-specific e.g., ‘Due to the medical treatment they are receiving’* | *?relationship with partners?*  *I would add about the following: (1) infant mortality – will my infant be okay? (2) long term outcomes – will the infant be impacted long term? Will my infant be negatively impacted or have delays or challenges due to being preterm? (3) worried about caring for my preterm infant when I am not in hospital – I will know what to do?*  *More general concerns about how to care for their baby – knowing if they are unwell*  *No*  *No*  *Not yet, …maybe fears for the future development of the baby*  *Scared to look after baby independently it feels to blame for the pre/mature birth* |
| I have felt unable to juggle motherhood with other responsibilities | 6 (37.5%) | *Ability to go through usual activities*  *Fine item for any mother*  *He talks about his concern about not being able to do everything he needs to do on a daily basis.*  *Including this item can be very pertinent because mothers of premature infants may experience heightened vulnerability due to the critical and intensive care needs of their babies. This can intensify feelings of anxiety, particularly about balancing caregiving with other responsibilities*  *The NICU experience would impact a mothers responsibilities and how they can manage everything* | 5 (31.3%) | *All feel this!*  *Normal reaction*  *Not prem-specific*  *These could be highly varied and don’t necessarily reflect anxious for the baby.* | 2 (12.5) | *Add which prevents me from doing necessary day to day (eating or personal hygiene)*  *Need to make prem-specific e.g., due to time commitments of my baby’s medical treatment.* |  |
| I have worried that my baby is picking up on my anxieties | 8 (50.0%) | *Exacerbated fears about infant wellbeing potentially negatively affecting the mother-infant relationship.*  *Expresses the mother’s overflowing anxiety*  *Fine item for any mother*  *Having a premature infant may cause more anxiety than a full-term infant, thus this make sense to keep*  *Parents often worry that their anxiety will impact baby due to the impact of the Neonatal unit*  *Self-assessment of anxiety*  *Sign of anxiety* | 2 (12.5%) | *Removing this item could be pertinent because it might introduce ambiguity into the screening process, as it relies heavily on the mother’s perception of her baby’s awareness and reactions, which can be influenced by numerous non-anxiety related factors, such as the baby’s general disposition or other environmental stimuli.* | 3 (18.8%) | *Need to make prem-specific e.g., because I have been so worried about the health of my pre-term baby*  *So they do not ask for help* |  |
| I have had negative thoughts about my relationship with my baby | 13 (81.3%) | *Bonding can be specifically impacted in the NNU and parents are reluctant to bond with the risk of loss*  *Bonding difficulties due to postnatal separation*  *Exacerbated guilt and shame for negative emotions due to infant’s critical condition*  *Fine item for any mother*  *Including this item can be very pertinent because mothers of premature infants may face particular challenges in bonding due to the baby’s initial medical fragility, intensive care needs, and possible physical separation after birth.*  *OCD*  *Parents could be stressed about their relationship with their baby*  *Reflect potential risk to baby*  *She expresses that she is afraid of not knowing how to take good care of her baby.*  *Sign of anxiety* | 0 (0.0%) |  | 1 (6.3%) | *Need to make prem-specific e.g., because I am worried about harm to prem baby, because I wish my baby were healthier/born well etc.* |  |
| I have worried about my baby’s milk intake | 10 (62.5%) | *Breastfeeding might be only one of (if not the only) way a mum can ‘do something’ for their baby in the best interest of their wellbeing. Control and morality and mental health relevance very high here*  *Feeding is a major issue*  *Fine item for any mother*  *Milk intake is highly monitored and discussed daily for premature babies*  *Preterm infants may eat differently than full-term, thus may cause anxiety*  *Related to fear of baby not growing enough*  *Talks about baby care concerns* | 3 (18.8%) | *Normal reaction*  *Removing this item can be pertinent because concerns about a baby’s milk intake are common among all parents, particularly those with newborns, and do not necessarily indicate anxiety. This worry is a typical part of infant care, focusing on ensuring that the baby is receiving adequate nutrition.*  *Usual worries when have a newborn* | 1 (6.3%) | *Need to make prem-specific e.g., due to the medical treatment they are receiving* |  |
| I have worried that my baby will stop breathing whilst sleeping | 15 (93.8%) | *Concern for basic activities*  *Fear of things out of control, infant perceived as vulnerable might die*  *Fine item for any mother*  *Including this item can be very pertinent because in case of premature babies, parental vigilance regarding breathing and other vital signs is often heightened due to past medical experiences (like NICU stays). This item helps to differentiate between normal vigilance and anxiety that may require intervention*  *Major anxiety during NICU and after*  *May be a higher likelihood of this happening*  *OCD and own sleep*  *Parents may be worried about the health of their infant*  *Sign of anxiety*  *Talks about baby care concerns*  *Unlikely outcome, if worried about it then why*  *Worries about infant harm and death probably elevated especially is separation required for medical reasons* | 0 (0.0%) |  | 1 (6.3%) | *Need to make prem-specific e.g., due to their prem status* |  |
| I have felt that I have had less control over my day than before my baby was born | 6 (37.5%) | *Fine item for any mother*  *He talks about his concern about not being able to do everything he needs to do on a daily basis.*  *Including this item can be very pertinent because for mothers of premature infants, these feelings can be intense due to the unpredictability and increased demands of caring for a medically vulnerable child*  *Often days are dictated by appointments*  *Parents of preterm infants, especially in the NICU, may feel like they aren’t in control*  *Very highly relevant due to uncertainties and lack of control over infant’s physical wellbeing.* | 3 (18.8%) | *Doesn’t seem central here*  *Normal reaction*  *To be expected if have a newborn* | 2 (12.5%) | *I would modify it to “less control over my life” to match more with mothers fear of something happening unexpectedly as a premature birth. As it could be common to many parents*  *Need to make prem-specific e.g., I have felt I have had less control due to my prem-baby’s health status/medical treatment etc.* |  |
| I have repeatedly checked on my sleeping baby | 14 (87.5%) | *Again, relevant due to uncertainty and lack of control over infant’s health status.*  *As above*  *Concern for basic activities*  *Fear of things out of control and that infant who is perceived as particularly vulnerable might die*  *Fine item for any mother*  *Including this item can be very pertinent because it may help to identify hyper-vigilance. For mothers of premature babies, who often have experienced real medical scares, this vigilance, although sometimes necessary, can become excessive and interfere with the mother’s ability to function normally.*  *Major issue as stated above.*  *Parents could be worried about their infant during sleep*  *Sleep*  *Talks about baby care concerns*  *Unlikely outcome anything wrong, why would you check repeatedly unless anxious?* | 0 (0.0%) |  | 1 (6.3%) | *Need to make prem-specific e.g., due to fears about how their prem status might affect their health* |  |
| I have felt that my baby would be better cared for by someone else | 11 (68.8%) | *Fear of not being a “good enough infant” as infant wasn’t kept safe inside mother long enough*  *Fine item for any mother*  *If parents are in the NICU, they may feel the nurses/doctors are better able to care for their infant than themselves*  *Not a normal response. Srong sign of anxiety*  *Reflect potential risk to the mother*  *Self dountb*  *Talks about baby care concerns*  *When babies have been cared for by nurses and medics parents can struggle to transition to be the primary care provider* | 2 (12.5%) | *Most mothers would not say that*  *This is inevitable. If baby was born prem and needs medical assistance, baby IS better cared for by someone else. It’s a medical necessity so I don’t think you’d be tapping into parenting anxieties here.* | 1 (6.3%) | *Remove item* |  |
| I have worried more about my finances than before my baby was born | 5 (31.3%) | *Financial strain of neonatal care*  *Fine item for any mother*  *If parents had to take more time off than expected, this may cause anxiety*  *Including this item can be very pertinent because financial stress is a known risk factor for developing anxiety disorders. Worrying about finances after the birth of a premature baby can exacerbate or trigger anxiety symptoms, making this a pertinent item for screening maternal anxiety.*  *She talks about day-to-day concerns and changes after childbirth.* | 4 (25.0%) | *Common anxiety for many parents*  *Not prem-specific.*  *Too multifactorial, this feels more a component of social stress rather than anxiety.* | 1 (6.3%) | *Remove item (not prem-specific, unless you frame as something like due to the number of hospital appointments my baby needs for medical treatment due to their prem-status).* |  |
| I have worried about my baby’s weight | 11 (68.8%) | *An infants weight is important for preterm infants, thus it makes sense that a parent is worries about their weight gain*  *Concern for basic wellbeing*  *Fear of baby not growing enough*  *Fine item for any mother, especially for premature infants*  *Major issue for preemies*  *Similar to the milk intake question. Concern about weight likely to be elevated in this group. Relevant anyway but then add in the fact that baby is likely poorly, these are behavioural aspects which the mum will be able to enact some perceived control over so likely to be driving mental health difficulties.*  *Talks about baby care concerns* | 1 (6.3%) | *Normal reaction* | 1 (6.3%) | *Need to make prem-specific e.g., due to their prem-status.* |  |
| I have worried that I am not going to get enough sleep | 6 (37.5%) | *A parent might be differently affected by sleep*  *Fine item for any mother*  *He talks about his concern about not being able to do everything he needs to do on a daily basis.*  *Including this item can be very pertinent because mothers of premature babies may worry more about the compounding effects of sleep loss on their ability to function and provide care.* | 2 (12.5%) | *Common for many parents*  *Not relevant here* | 2 (12.5%) | *I would modify to I have not had enough sleep because of my worries.*  *Need to make prem-specific e.g., due to their prem-status, medical treatments, lack of routine, worry about infant health (needs to be more specific).* |  |
| I have felt frightened when my baby is not with me | 12 (75.0%) | *Concern for babies wellbeing.*  *Due to the fragile nature*  *Fear of being out of control and that an infant perceived as particularly vulnerable is going to die*  *Fine item for any mother*  *For different reasons to a healthy birth. I think something needs to be in here about ‘in case something bad happens’ to make more prem-specific.*  *Including this item can be very pertinent because it identifies separation anxiety, which is common among new mothers, particularly those of premature babies who may have already experienced significant stress due to early infant separation if their infant required NICU care.*  *Separation anxiety is central*  *Sign of anxiety*  *Talks about baby care concerns* | 0 (0.0%) |  | 1 (6.3%) | *Need to make prem-specific e.g., why? Risk of death? Risk of harm during care?* |  |
| I have worried about getting my baby into a routine | 4 (25.0%) | *Due to disruptions with medical treatment etc. The items need re-wording to make more prem-specific.*  *Fine item for any mother* | 4 (25.0%) | *All new parents worry about routines*  *Common for many parents*  *Normal reaction* | 2 (12.5%) | *I am not sure about the modification of item 14 because it expresses worries but they are general worries about the routine*  *Need to make prem-specific e.g., due to disruption of medical treatment?* |  |
| I have worried about my baby being accidentally harmed by someone or something else | 10 (62.5%) | *Due to medical treatments*  *Due to the stress of many people care for infant in the NICU*  *Fine item for any mother*  *Including this item can be very pertinent because anxiety can manifest as an increased protective vigilance, where mothers might be overly concerned about potential dangers affecting their baby*  *Sign of anxiety*  *Talks about baby care concerns* | 0 (0.0%) |  | 1 (6.3%) | *Need to make prem-specific e.g., during medical care? Due to their prem-status?* |  |
| I have worried I will not know what to do when my baby cries | 12 (75.0%) | *All babies cry – worrying about excessively demonstrates anxiety*  *Concern about their relationship with their baby*  *Discusses concerns about her baby*  *Due to seapration during medical treatments, loss of confidence during periods of separation etc.*  *Feelings of being less competent are often related to premature birth*  *Fine item for any mother*  *Parent may not know what is wrong and may not be able to give appropriate care, especially if in the NICU* | 1 (6.3%) | *Common for many parents* | 1 (6.3%) | *Need to make prem-specific e.g., during medical care? Due to their prem status?* |  |
